# Interleukin-4 Receptor Alpha Expressing B Cells Are Essential to Down-Modulate Host Granulomatous Inflammation During Schistosomasis

**DOI:** 10.3389/fimmu.2018.02928

**Published:** 2018-12-18

**Authors:** Hlumani Ndlovu, Justin Komguep Nono, Nada Abdel Aziz, Natalie Eva Nieuwenhuizen, Frank Brombacher

**Affiliations:** ^1^International Center for Genetic Engineering and Biotechnology (ICGEB), Cape Town Component, Cape Town, South Africa; ^2^Division of Immunology, Institute of Infectious Diseases and Molecular Medicine (IIDMM), University of Cape Town, Cape Town, South Africa; ^3^South African Medical Research Council (SAMRC), Immunology of Infectious Disease Research Unit, Cape Town, South Africa; ^4^Department of Integrative Biomedical Sciences, Faculty of Health Sciences, University of Cape Town, Cape Town, South Africa; ^5^The Medical Research Centre, Institute of Medical Research and Medicinal Plant Studies (IMPM), Ministry of Scientific Research and Innovation, Yaounde, Cameroon; ^6^Chemistry Department, Faculty of Science, Cairo University, Cairo, Egypt

**Keywords:** schistosomiais, B cells, pathology, IL-4RA, chronic infection

## Abstract

Schistosomiasis (bilharzia) is a parasitic helminth disease that can cause severe inflammatory pathology leading to organ damage in humans. Failure of the host to regulate egg-driven granulomatous inflammation causes host morbidity during chronic infection with *Schistosoma mansoni*. Although the importance of B cells in regulating pathology during chronic infection has been well defined, the specific contribution of IL-4Rα-expressing B cells is still unknown. To address this, we examined B cell-specific IL-4Rα-deficient (mb1^cre^IL-4Rα^−/lox^) mice in three experimental models of schistosomiasis: high-dose (100 cercariae), low dose (30 cercariae), and a synchronous egg challenge. In the high dose model, we found that mice deficient in IL-4Rα-expressing B cells were more susceptible to acute schistosomiasis than B cell-deficient (μMT) mice, succumbing to infection at the acute stage whereas μMT mice survived until the chronic stage. An *S. mansoni* egg challenge model demonstrated that deleting IL-4Rα expression specifically on B cells resulted in increased lung granulomatous pathology, suggesting a role for this B cell subset in controlling granulomatous pathology. In agreement with this, a low dose model of schistosomiasis—which mimics the course of clinical chronic disease—demonstrated that depleting IL-4Rα-expressing B cells in mb1^cre^IL-4Rα^−/lox^ mice considerably impaired the host ability to down-modulate granulomatous inflammation in the liver and gut during chronic schistosomiasis. Taken together, our findings indicate that within the B cell compartment, IL-4Rα-expressing B cells in particular down-modulate the deleterious egg-driven tissue granulomatous inflammation to enable host survival during schistosomiasis in mice.

## Introduction

The ability of B cells to drive host protective defense mechanisms during parasitic infections has received a lot of attention of late. Studies from over two decades ago utilized B cell deficient mice (μMT) that were generated by targeting the IgM transmembrane domain, resulting in the impairment of the B cell compartment. B cell-deficient mice were found to be susceptible to schistosomiasis, succumbing to infection at the chronic stage of disease, and displaying augmented liver granulomatous pathology (1, 2). Moreover, B cells have been shown to be crucial for the development of host protective effector and memory CD4^+^ T cells responses to *Pneumocystis* lung infection (3). In contrast, B cells are dispensable for driving host protective immunity to infection with the intracellular parasite *Leishmania major* (*L. major*), with B cell-deficient mice developing intermediate resistance to the infection between Balb/c and C57BL6 mice (4).

The general contribution of B cells to host immunity against infection is well established, and recent studies focus on specific subsets of B cells and B cell-derived effector molecules. A pioneering study by Harris et al. classified B cells into two effector subsets: B effector 1 (Be1) cells that secrete IFN-γ, IL-12p40, and TNF-α under the control of the transcription factor *T-bet* (5, 6) and B effector 2 (Be2) cells that produce low IL-4, IL-13, and IL-2 after receiving instruction from IL-4, IL-4Rα, and Th2 cells (5, 7, 8). The latter subset was identified *in vitro* and *in vivo* after infection with *H. polygyrus*. We have recently shown that IL-4-producing B cells influence T helper cell dichotomy within the first 3 days of infection in the lymph node, which leads to a host protective type 2 immune response during acute schistosomiasis but is detrimental to the host during cutaneous leishmaniasis caused by *L. major* (9). Moreover, B cell-derived IL-2, and TNF-α are crucial for clearance of *Heligmosomoides polygyrus* (*H. polygyrus*) worms, development of CD4^+^ T cells secreting IL-4 and generation of type 2 antibody responses (10). Another key molecule derived from B cells that is crucial for the development of robust host defense mechanisms is the MHCII molecule, and mice carrying a specific deletion of MHCII on B cells failed to clear *H. polygyrus* infection and exhibited impaired humoral and cellular immunity (10).

Schistosomiasis is an important parasitic disease that affects more than 200 million people worldwide and is estimated to cause approximately 280 000 deaths per year in sub-Saharan Africa alone (11–14). The disease is caused by trematode flukes of the genus *Schistosoma*; mainly, *Schistosoma mansoni* (*S. mansoni*), *S. japonicum* and *S. haematobium*, which are infective to humans (11–13). The disease is driven by the thousands of eggs that become trapped in host tissues such as the liver, kidneys and intestines, triggering a robust immune-mediated granulomatous inflammation. This causes local and systemic manifestations like anemia, growth stunting, impaired cognition, hepatosplenomegaly, periportal fibrosis, urogenital inflammation, and scarring that ultimately lead to host morbidity and eventual death in some severe cases (11). The immune response is characterized by a triphasic kinetic, with phase 1 dominated by a Th1 response induced by worm antigens, phase 2 (acute stage) characterized by an egg-driven, highly polarized Th2 granulomatous response, and immunomodulatory responses occurring in phase 3 (chronic stage) (15, 16).

Earlier studies aimed at elucidating the immunological factors driving host protective immunity to schistosomiasis took advantage of constitutive gene-deficient mouse models. These studies demonstrated that T and B cells (17–21) and Th2 effector molecules (IL-4, IL-13, IL-10, IL-4Rα, STAT-6) are crucial for conferring host protective immunity to infection (22–31). In our laboratory, we have looked in more detail at the cell-specific requirements of IL-4Rα in driving host survival during *S. mansoni* infection. We have found that IL-4Rα signaling on macrophages and neutrophils (32), smooth muscle cells (33), and pan-T cells (34) individually contribute to driving host protective immunity and down-modulating excessive tissue pathology during acute schistosomiasis.

For the host to survive the chronic stage of *S. mansoni* infection, the dominant Th2 immune response driven by the eggs needs to be down-regulated to enable the host to control the fibrogranulomatous damage (1, 35–40). Immuno-suppressive CD8^+^ T cells (36), cross-regulation by cytokines produced by Th1 or Th2 cells (26, 37, 38) and FcR signaling on B cells (1, 2) have all been implicated in the immunomodulatory mechanisms required to ameliorate tissue pathology during chronic schistosomiasis. The immunomodulatory role of B cells was accidentally demonstrated in a study that targeted IL-10R using antibodies and found that B cells were depleted in the liver with a consequent augmented pulmonary granulomatous pathology during chronic schistosomiasis (41).

In this study, we investigated the immunomodulatory role of IL-4Rα expressing B cells during schistosomiasis. Using transgenic mice that lacked IL-4Rα expression on B cells (*mb1*^cre^IL-4Rα^−/lox^), we showed that mice lacking this subset of B cells are susceptible to schistosomiasis, succumbing to disease earlier than both littermate controls and global B cell deficient mice. We found that the lack of IL-4Rα expressing B cells resulted in augmented granulomatous pathology in the liver and gut, and a profound inflammatory response characterized by increased concentrations of IL-4, IL-5, IL-6, IFN-γ, and IL-17 at the later stages of chronic schistosomiasis infection. Finally, *in situ* analysis revealed that mice lacking B cell-specific IL-4Rα expression failed to down-regulate granulomatous lung pathology after synchronous *S. mansoni* eggs challenge. Taken together, these findings demonstrated that IL-4Rα expressing B cells play a crucial immunomodulatory role that limits T cell responses and granulomatous tissue pathology during chronic schistosomiasis.

## Results

### Mice Lacking IL-4Rα Expressing B Cells Are More Susceptible to Schistosomiasis Than B Cell-Deficient Mice

**Figure 1:**
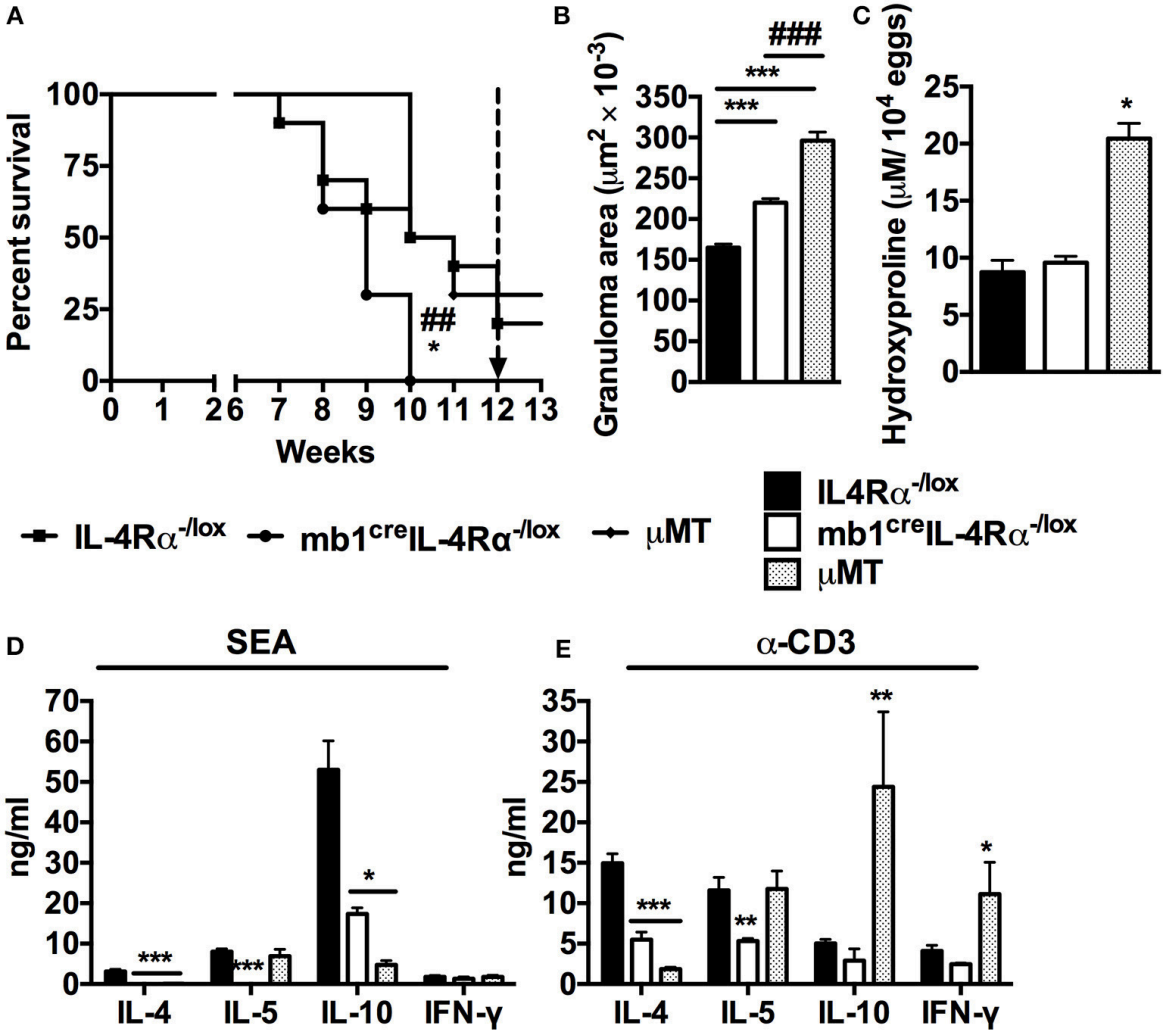
Mice lacking IL-4Rα expressing B cells succumb to schistosomiasis by 10 weeks post-infection. IL-4Rα^−/lox^, *mb1*^cre^IL-4Rα^−/lox^ and μMT mice were infected with 100 *S. mansoni* cercariae and monitored weekly. **(A)** Survival kinetics of mice infected with *S. mansoni* (*n* = 8–10 mice). Survival curves were compared using Logrank test. *p < 0.05 and **p < 0.01 vs. IL-4Rα^−/lox^ mice. **(B)** Granuloma area measured by microscopic analysis of formalin-fixed liver sections after H&E staining. **(C)** Liver fibrosis measured as hydroxyproline content normalized to tissue egg numbers (mean ± SEM, *n* = 4–6). **(D)** Cytokine production by total mesenteric lymph node cells restimulated with either SEA. **(E)** Cytokine production by total mesenteric lymph node cells restimulated with α-CD3 (mean ± SEM, *n* = 8–10 mice). Data are representative of two independent experiments. *p < 0.05, **p < 0.01 and ***p < 0.001 vs. IL-4Rα^−/lox^ mice.

A previous study showed that mice deficient in B cells displayed heightened susceptibility to schistosomiasis at the chronic stages of infection (1, 2). Moreover, we have recently shown that *mb1*^cre^IL-4Rα^−/lox^ mice, which lack IL-4Rα expression specifically on B cells, are highly susceptible to acute schistosomiasis and display increased hepatocellular damage (9). To investigate whether IL-4Rα expressing B cells contribute to the B-cell mediated host resistance to schistosomiasis, we infected *mb1*^cre^IL-4Rα^−/lox^, μMT and IL-4Rα^−/lox^ mice with 100 live *S. mansoni* cercariae and monitored them over a 13 weeks period. *Mb1*^cre^IL-4Rα^−/lox^ mice began to die at 7 weeks post-infection and they had all succumbed to infection by 10 weeks post-infection compared to littermate control mice that had 50% survival at the same time point (Figure 1A). In contrast, B cell-deficient (μMT) mice displayed delayed susceptibility when compared to *Mb1*^cre^IL-4Rα^−/lox^ mice, with mice surviving the acute stage of infection (Figure 1A). However, during the chronic stage of infection, B cell deficient mice had a drastic increase in mortality rate, with 50% of mice succumbing to infection within the same week (Figure 1A). Taken together, these data suggest that the specific deficiency of IL-4Rα expressing B cells is equally if not more deleterious to the host than the general lack of B cells during acute schistosomiasis.

Liver granuloma formation was compared between *mb1*^cre^IL-4Rα^−/lox^ mice and μMT mice at 7 weeks post *S. mansoni* infection. As observed previously, B cell-specific IL-4Rα deficient mice had augmented granuloma size compared to littermate control mice (Figure 1B). However, the granulomas from B cell deficient mice were larger than the granulomas from B cell-specific IL-4Rα deficient mice (Figure 1B). In fact, B cell deficient mice developed granulomas that were almost twice the size of those from littermate IL-4Rα^−/lox^ mice (Figure 1B) confirming a central role for B cells in the control of the host granulomatous response during schistosomiasis. However, livers from μMT mice contained almost double the concentration of hydroxyproline than both the *mb1*^cre^IL-4Rα^−/lox^ and littermate control mice during the acute phase of infection, indicating increased hepatic fibrosis in the absence of total B cells but not specifically IL-4Rα expressing B cells (Figure 1C). These data suggest that whereas IL-4Rα expressing B cells contribute to the control of the liver granulomatous response during schistosomiasis, other unidentified B cell subset(s) is(are) similarly important. Intriguingly, however, our findings also indicate that B cells mediate the down-regulation of hepatic fibrosis in this context independently from IL-4Rα expressing B cells.

We further restimulated MLN cells from infected mice with either *Schistosoma* egg antigen (SEA) or α-CD3 *in vitro* and measured cytokine production by ELISA. In accordance with our recent report (9), cells from *mb1*^cre^IL-4Rα^−/lox^ mice failed to produce the Th2 cytokines IL-4, IL-5, and IL-10 in response to antigen-specific stimuli compared to littermate control mice (Figure 1D). Similarly, cells from μMT mice stimulated with SEA failed to produce IL-4 and IL-10 although the levels of IL-5 were the same as those in littermate control mice (Figure 1D). Conversely, mitogenic stimulation of cells from μMT mice triggered a substantial release of IL-10 and IFN-γ while the production of IL-4 was diminished compared to littermate control mice (Figure 1E). Finally, in comparison to littermate control mice, cells from B cell-specific IL-4Rα deficient mice showed defects in the production of IL-4 and IL-5 while the production of IL-10 and IFN-γ was unaltered after restimulation with α-CD3 (Figure 1E). In as much as our data unveil a differential immune responsiveness between cells from μMT and *mb1*^cre^IL-4Rα^−/lox^ mice when compared to littermate control IL-4Rα^−/lox^ mice, they demonstrate a similar need for total B cells and IL-4Rα expressing B cells in driving the development of optimal Th2 responses during *S. mansoni* infection *in vivo*.

To explore the impact of IL-4Rα deficiency on B cells on the differentiation and cytokine production by CD4^+^ T cells during infection, single cell suspension was prepared from MLN and cells were stained for flow cytometry analysis. There was no significant difference in the absolute number of CD3^+^CD4^+^ T cells present in the MLN in all mutant mouse strains (Figure S1A). However, the lack of IL-4Rα expression on B cells significantly hindered the differentiation of CXCR5^+^ T_FH_ cells (Figure S1B) and effector CD4^+^ T cells (CD4^+^CD44^hi^D62L^lo^, Figure S1C) compared to littermate control mice. Similarly, B cell deficient mice exhibited reduced numbers of CXCR5^+^ T_FH_ cells and effector CD4^+^ T cells compared to littermate control mice (Figures S1B,C). Examination of intracellular cytokine production by CD4^+^ T cells restimulated with PMA/Ionomycin *ex vivo* revealed that abrogation of IL-4Rα expression on B cells results in reduced production of Th2 cytokines IL-4 and IL-13 albeit IFN-γ production was not altered compared to control mice (Figure S1D). Likewise, CD4^+^ T cells from μMT mice failed to produce Th2 cytokine IL-4 and IL-13 after *ex vivo* restimulation with PMA/Ionomycin compared to littermate control mice (Figure S1D). Therefore, IL-4Rα expression on B cells is crucial for differentiation of CD4^+^ T cells and generation of CD4^+^ Th2 immunity.

B cells have been shown to produce cytokines in response to antigen-specific stimulation or *in vivo* during infection (13). To explore the ability of IL-4Rα deficient B cells to produce cytokines during *S. mansoni* infection, single cell suspension was prepared from MLN and cells restimulated with PMA/Ionomycin before intracellular cytokine detection by flow cytometry analysis. The absolute number of CD19^+^B220^+^ B cells was comparable between both mutant strains (Figure S1E). However, the total number of follicular B cells (B220^+^CD21^hi^CD23^hi^) was significantly reduced in *mb1*^cre^IL-4Rα^−/lox^ mice (Figure S1F), while the number of marginal zone B cells was significantly increased compared to littermate control mice (Figure S1G). We observed a general abrogation of cytokine producing B cells in B-cell-specific IL-4Rα-deficient mice compared to littermate control mice (Figure S1H). Therefore, these data suggests that B cell-specific IL-4Rα expression is required for initiating expression of type 1 and type 2 cytokine during helminth infection.

### IL-4Rα Expressing B Cells Are Required for Containment of Granulomatous Pathology During Chronic Schistosomiasis

**Figure 2:**
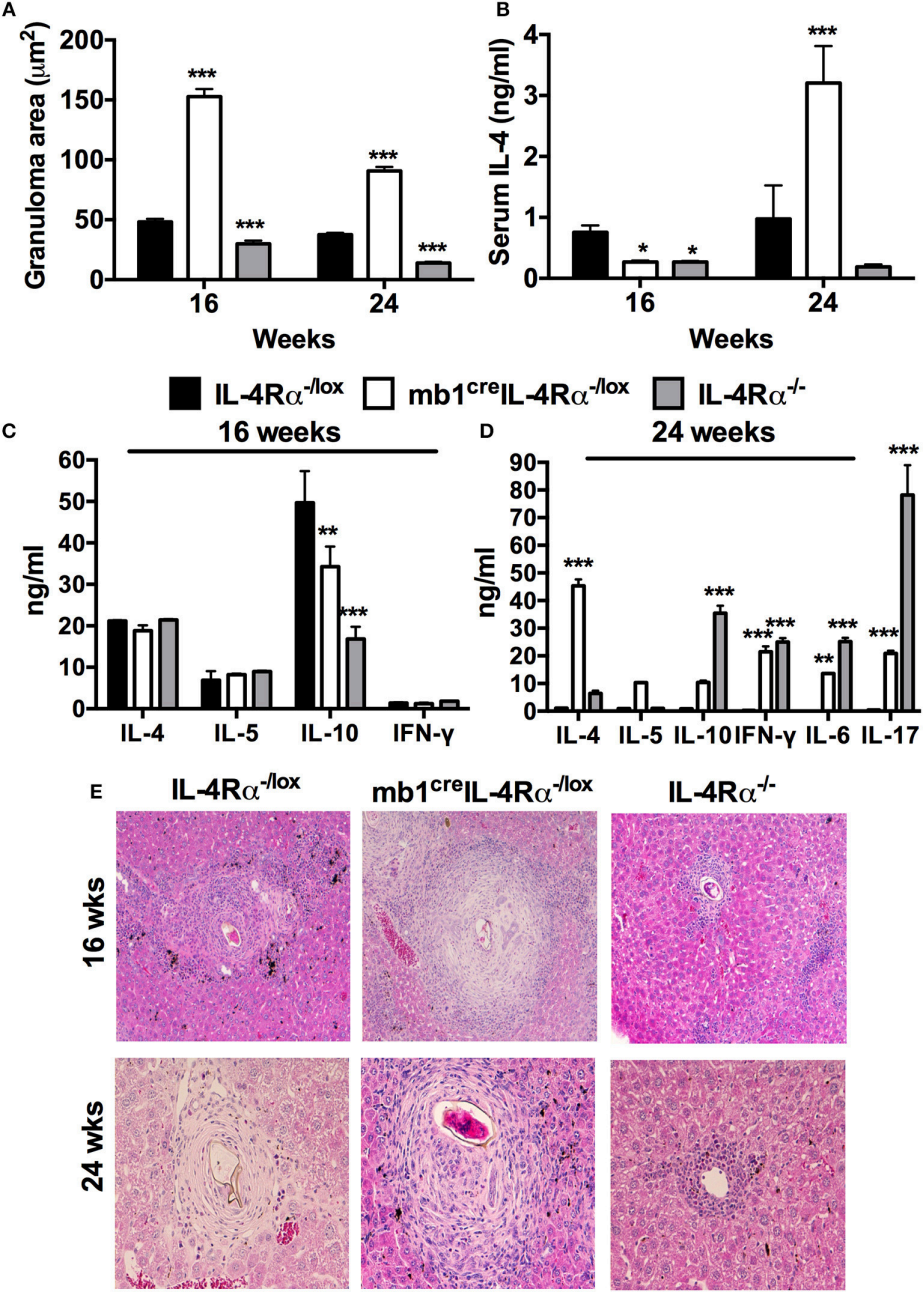
IL-4Rα expressing B cells are required to down-regulate hepatic pathology during chronic schistosomiasis. IL-4Rα^−/lox^, *mb1*^cre^IL-4Rα^−/lox^, and IL-4Rα^−/lox^ mice were infected with 30 *S. mansoni* cercariae and killed at 16 and 24 weeks post-infection. **(A)** Liver granuloma area was measured using a computerized morphometric analysis program (NIS elements by NIKON) by measuring 20–25 granulomas per mouse. **(B)** Serum IL-4 levels were detected by ELISA at both time points. **(C,D)** Detection of cytokine production by total MLN cells after *in vitro* restimulation with α-CD3 for 72 h. **(E)** Histology images showing liver granuloma formation at 16 and 24 weeks post-infection. Data represent two independent experiments. *p < 0.05, **p < 0.01, ***p < 0.001 vs. IL-4Rα^−/lox^ mice. *n* = 4–6 mice per group.

To further assess the suggested importance of IL-4Rα expression on B cells for the down-regulation of *S. mansoni* egg-driven fibrogranulomatous inflammation, we performed a low dose infection of *mb1*^cre^IL-4Rα^−/lox^ mice (30 live *S. mansoni* cercariae) and killed mice at 16 (chronic) and 24 (advanced chronic) weeks post-infection to analyse tissue pathology and immune profiles of chronically infected mice. We did not observe any significantly different mortality between the mutant strains during the entire course of chronic *S. mansoni* infection (Table S1). Mice lacking IL-4Rα expressing B cells had significantly enlarged granulomas in the liver at both 16 and 24 weeks post-infection compared to littermate control mice (Figures 2A,E). Interestingly, although the levels of serum IL-4 were significantly reduced in infected *mb1*^cre^IL-4Rα^−/lox^ mice at 16 weeks post-infection, the levels of IL-4 were almost 3-fold higher in mice lacking IL-4Rα expressing B cells at 24 weeks post-infection (Figure 2B). Analysis of the cytokine profile produced by total MLN cells restimulated with 20 μg/ml of α-CD3 revealed that the production of IL-10 was significantly decreased in *mb1*^cre^IL-4Rα^−/lox^ mice compared to littermate control mice while the concentrations of IL-4, IL-5, and IFN-γ were similar in all mutants at 16 weeks post-infection (Figure 2C). In contrast, *mb1*^cre^IL-4Rα^−/lox^ mice developed significantly increased levels of IL-4, IL-10, IL-6, IL-17, and IFN- γ compared to IL-4Rα^−/lox^ littermate control mice at 24 weeks post infection (Figure 2D). Therefore, the data strongly suggested that IL-4Rα expressing B cells are required to down-regulate both hepatocellular damage and general cytokine responses during the late stages of chronic schistosomiasis.

We also investigated whether IL-4Rα expressing B cells were required to downregulate gut pathology during the chronic stages of schistosomiasis. Infected *mb1*^cre^IL-4Rα^−/lox^ mice developed large granulomas in the small intestines at 16 weeks post-infection and these became even larger at 24 weeks post-infection (Figure S2). In contrast, littermate control mice were able to modulate gut granulomatous pathology as indicated by the presence of small granulomas characterized by minor infiltration of immune cells (Figure S2). We also observed a comparable number of *S. mansoni* eggs shunted into the lungs in both *mb1*^cre^IL-4Rα^−/lox^ mice and IL-4Rα^−/lox^ littermate control mice at both 16 and 24 weeks post-infection (Figure S3) Therefore, these data demonstrate that IL-4Rα expressing B cells are essential for down-regulating gut granulomatous pathology during chronic schistosomiasis in mice.

### IL-4Rα Expressing B Cells Are Required to Control Lung Granulomatous Pathology During Synchronous *S. Mansoni* egg Challenge

**Figure 3:**
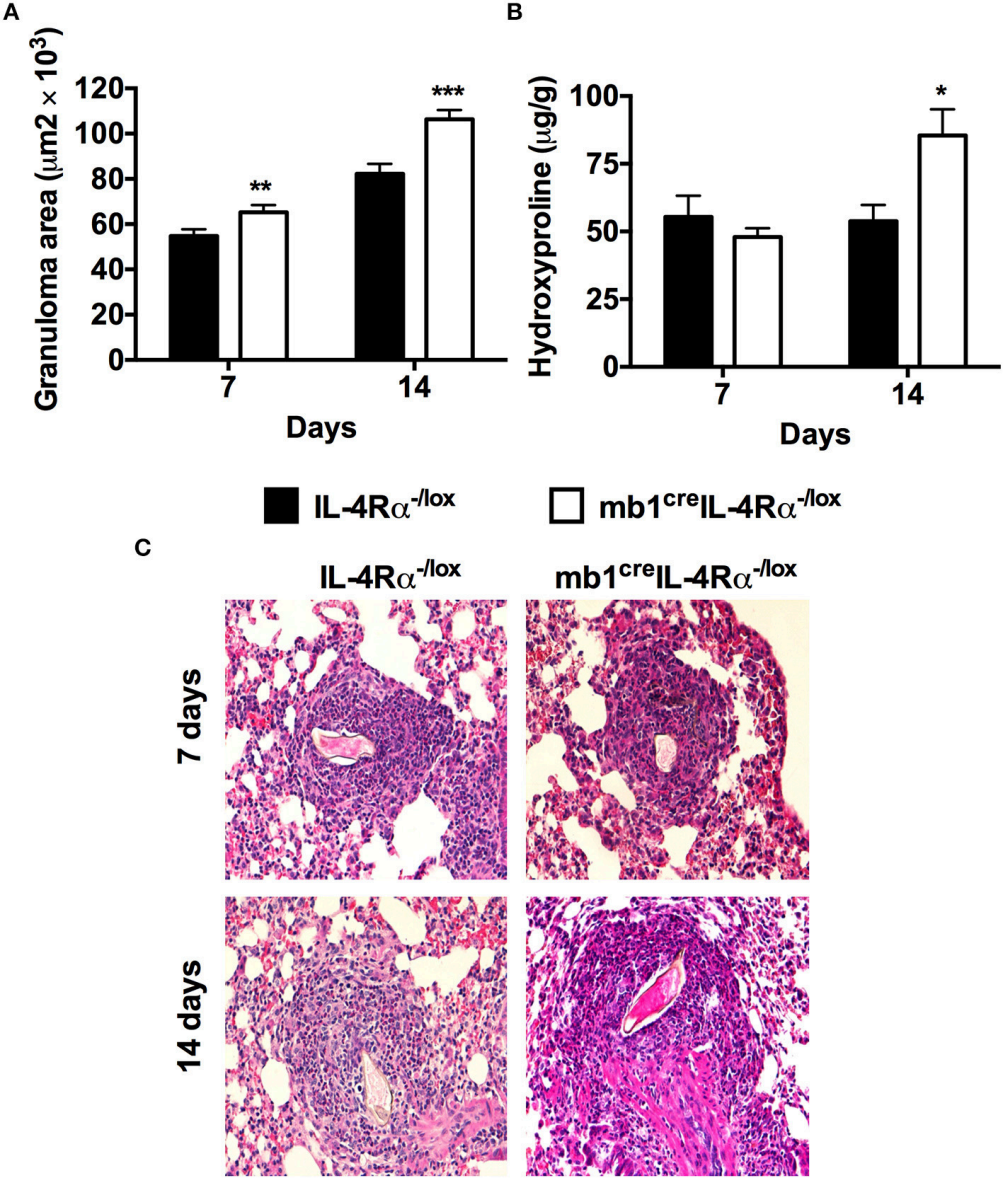
Mice lacking IL-4Rα expressing B cells fail to down-regulate early granulomatous pathology in the lungs after synchronous *S. mansoni* eggs challenge. IL-4Rα^−/lox^ and *mb1*^cre^IL-4Rα^−/lox^ mice were sensitized with 2 500 *S. mansoni* eggs intraperitoneally, challenged with 2 500 eggs intravenously 14 days later and killed over two time points (7 and 14 days post-challenge). **(A)** Granuloma formation was measured using a computerized morphometric analysis program (NIS elements by NIKON) by measuring 20–25 granulomas per mouse. **(B)** Lung fibrosis measured by determining hydroxyproline concentration. **(C)** Histological examination of H&E stained lungs sections. Data represent two independent experiments. *p < 0.05, **p < 0.01 and ***p < 0.001 vs. IL-4Rα^−/lox^ mice. *n* = 6 mice.

Since we had observed shunting of eggs to the lungs in both *mb1*^cre^IL-4Rα^−/lox^ and IL-4Rα^−/lox^ mice, we used a synchronous *S. mansoni* egg model where mice were first sensitized then challenged with 2,500 *S. mansoni* eggs to validate the effect of IL-4Rα expressing B cells on *S. mansoni* egg-driven fibrogranulomatous responses. Comparatively testing *mb1*^cre^IL-4Rα^−/lox^ and littermate IL-4Rα^−/lox^ mice, we found that the absence of IL-4Rα expressing B cells in *mb1*^cre^IL-4Rα^−/lox^ mice led to significantly increased lung granuloma areas at both 7 (65.271 ± 22.787 vs. 57.97 ± 21.099) and 14 days (106.388 ±29.590 vs. 82.252 ± 31.763) post-challenge compared to IL-4Rα^−/lox^ littermate control mice. (Figures 3A,C). The concentration of hydroxyproline was similar between all mutants at 7 days post-challenge, however, mb1^cre^IL-4Rα^−/lox^ mice had significantly increased fibrosis as indicated by high concentrations of hydroxyproline at 14 days post-challenge compared to littermate control mice (Figure 3B). Therefore, we can conclude that IL-4Rα responsive B cells are required to down-regulate granulomatous pathology and lung fibrosis during synchronous *S. mansoni* egg challenge.

### Impaired Secretion of Cytokines by B Cells Deficient in IL-4Rα Signaling.

**Figure 4:**
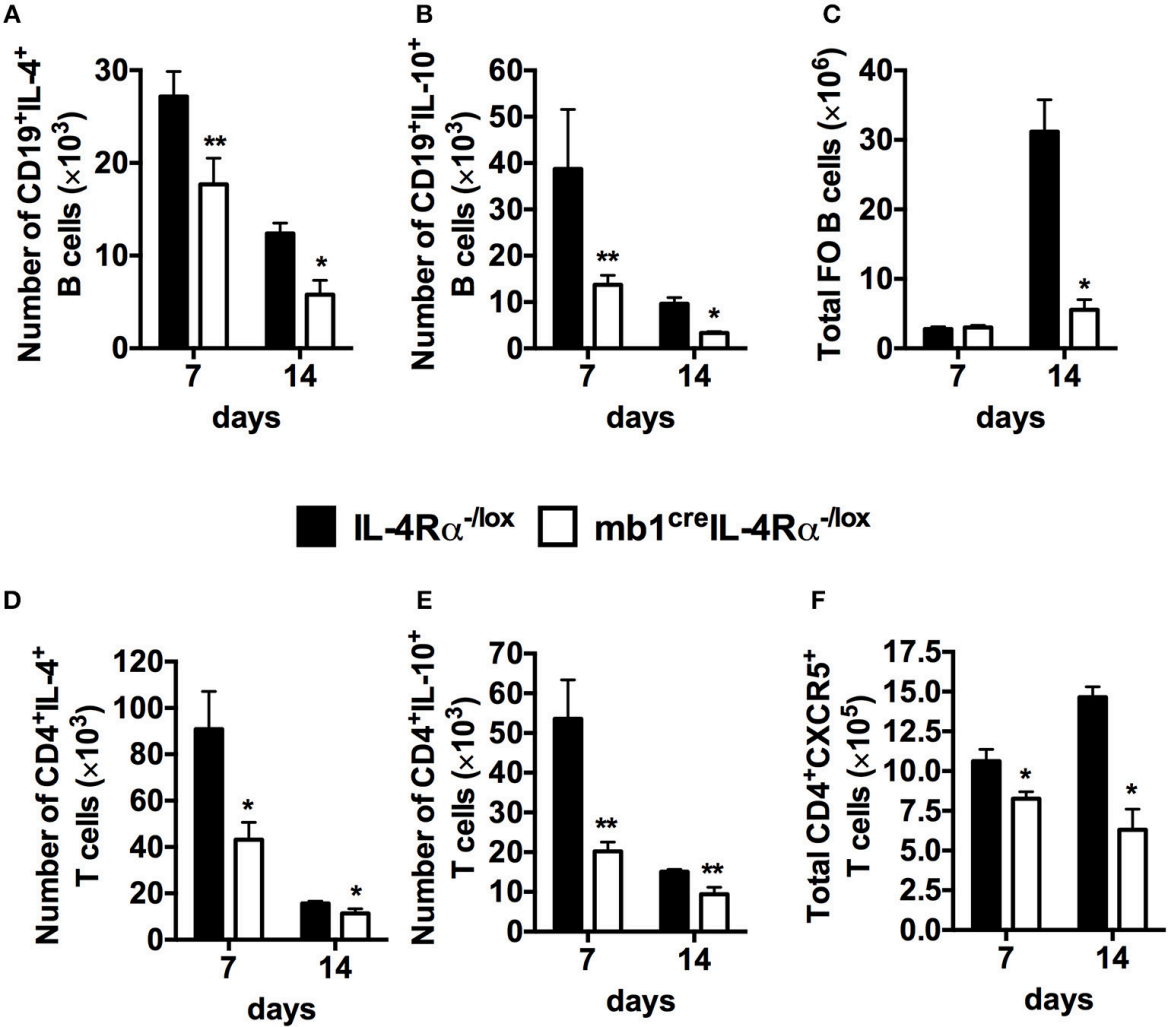
Abrogated cellular immunity in mice lacking IL-4 producing B cells after synchronous *S. mansoni* eggs challenged. Single cell suspensions were prepared from mediastinal lymph nodes (MST) and cells were stained for flow cytometry. **(A,B)** Intracellular cytokine detection after stimulating total MST cells with 50 ng/ml PMA and 250 ng/ml ionomycin *in vitro*. **(C)** Total number of follicular B cells (FO, CD19^+^CD23^hi^CD21^hi^) cells recruited to the mediastinal lymph node (MST). **(D,E)** Total number of CD4^+^ T cells producing IL-4 and IL-10 in the MST. **(F)** Total number of CXCR5^+^ T follicular helper (T_FH_) cells in the lung draining lymph nodes. Data are representative of two independent experiments. *p < 0.05, **p < 0.01 vs. IL-4Rα^−/lox^ mice. *n* = 6 mice per group.

Having defined a critical role for IL-4Rα expressing B cells in controlling the immune response and tissue pathology in response to *S. mansoni* eggs, we proceeded to investigate the immunological changes within the B cell population as a result of IL-4Rα removal which might explain the observed changes. Using the synchronous *S. mansoni* eggs challenge model, we analyzed cytokine secretion by CD19^+^ B cells after restimulation with phorbol myristate (PMA) and ionomycin. The number of B cells producing IL-4 and IL-10 in the mediastinal lymph nodes was significantly reduced in mb1^cre^IL-4Rα^−/lox^ mice both at 7 and 14 days post *S. mansoni* egg challenge compared to littermate control mice (Figures 4A,B, Figure S4). We also found that the number of follicular B cells (CD21^hi^CD23^hi^) was reduced at 14 days post *S. mansoni* egg challenge in B cell-specific IL-4Rα deficient mice compared to littermate control mice (Figure 4C, Figure S4). Moreover, examination of intracellular cytokine production by CD4^+^ T cells restimulated with PMA/ionomycin *ex vivo* revealed that abrogation of IL-4Rα expression on B cells indirectly resulted in significantly reduced number of CD4^+^ T cells producing IL-4 (Figure 4D, Figure S5), IL-10 (Figure 4E, Figure S5) and follicular helper T cells (T_FH_) expressing CXCR5 (Figure 4F, Figure S5) compared to littermate control mice at both time points (7 and 14 days post eggs challenge). Taken together, these data suggest that the absence of IL-4Rα from the B cell population impairs type-2 cytokine production by B cells, diminishes Th2 responses and Tfh cell expansion resulting in a severely diminished pool of follicular B cells in the lung-draining mediastinal lymph nodes of mb1^cre^IL-4Rα^−/lox^ mice after synchronous *S. mansoni* eggs challenge.

### The Lack of B Cell-Derived IL-4 Impairs Development of Th2 Responses in *S. Mansoni* Infected B-IL-4^−/−^ Mixed Bone Marrow Chimeras

We had previously demonstrated that sorted B cells from mb1creIL-4Rα-/lox mice displayed reduced IL-4 gene expression at day 4 post-challenge with *S. mansoni* eggs in the footpad compared to littermate control mice. To validate our observation that deletion of IL-4Rα on B cells impairs the development of optimal Th2 responses after *S. mansoni* infection by diminishing IL-4 and IL-10 production by B cells, we generated mixed bone marrow chimeras with a specific deficiency of B cell derived IL-4 (Figure S6). Here, sub-lethally irradiated B cell-deficient mice (μMT) were reconstituted with 50% μMT and 50% IL-4^−/−^ bone marrow (BM) to generate mixed bone marrow chimeras that lacked IL-4 production specifically on B cells (B-IL-4^−/−^, Figure S6). As a control, recipient mice were reconstituted with 100% Balb/c BM to generate wild-type chimeras (WT), sufficient in IL-4 production in all hematopoietic cells (Figure S6). Finally, recipient mice were reconstituted with 100 IL-4^−/−^ BM to generate chimeras that had impaired IL-4 production in all hematopoietic cells (IL-4^−/−^, Figure S6). All the bone marrow chimeras contained equivalent proportions of CD3^+^CD4^+^ T cells (Figure S7A, CD19^+^B220^+^ B cells (Figure S7B), CD11b^+^ cells (Figure S7C), and CD11c^+^ cells (Figure S7D) in peripheral blood 8 weeks after reconstitution, indicating successful reconstitution.

The efficiency of reconstitution was further confirmed by analyzing antibody responses in sera of mixed bone marrow chimeras that were infected with 100 live *S. mansoni* cercariae and killed 7 week post-infection. WT and B-IL-4^−/−^ chimeras had similar titers of SEA-specific IgG1 and total IgE antibodies after *S. mansoni* infection (Figures S8A,B). Conversely, WT and B-IL-4^−/−^ chimeras failed to produce antigen specific type 1 (IgG2a and IgG2b) antibody isotypes (Figures S8C,D). Infected IL-4^−/−^ chimeras failed to switch the class of antibody isotypes as demonstrated by high titers of antigen specific type 1 antibody isotypes (IgG2a and IgG2b) and reduced type 2 antibody isotypes (Figures S8A–D). These data demonstrated that mice developed sufficient type 2 antibody titers in response to *S. mansoni* infection independently of B cell-derived IL-4.

**Figure 5:**
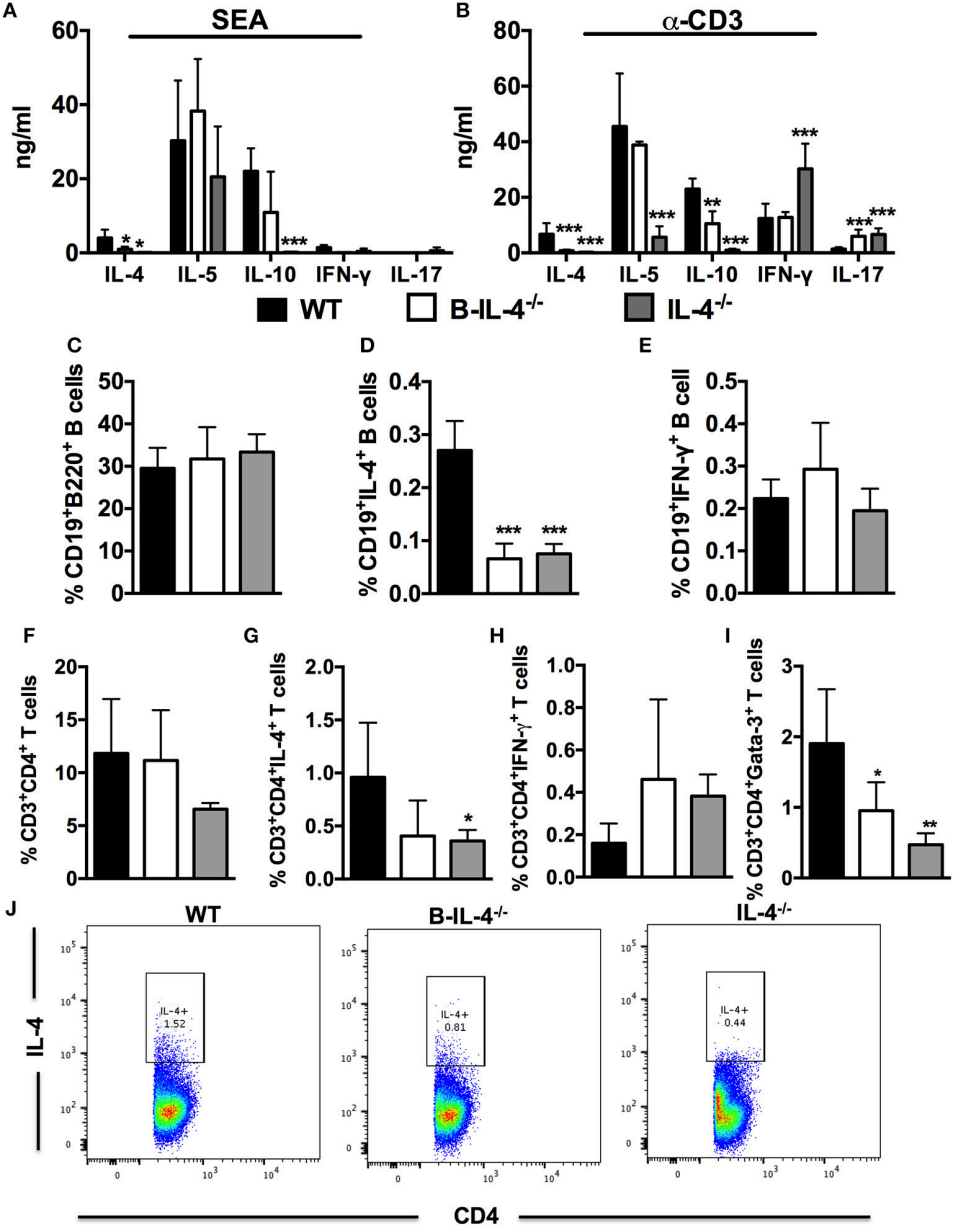
Impaired Th2 immunity in mice lacking IL-4 producing B cells. Bone marrow chimeras were infected with 100 live *S. mansoni* cercariae and killed 7 weeks post-infection. Single cell suspensions were prepared from MLN and cells were restimulated with 20 μg/ml SEA or α-CD3 *in vitro*. **(A,B)** Cytokine production by restimulated total MLN cells was detected by ELISA. **(C)** Frequency of CD19^+^B220^+^ B cells in the gut draining lymph node. **(D,E)** Detection of intracellular cytokines produced by CD19^+^ B cells after restimulation of total MLN cells with 50 ng/ml PMA and 250 ng/ml ionomycin. **(F)** Frequency of CD3^+^CD4^+^ T cells in the MLN. **(G,H)** Intracellular cytokine production by CD3^+^CD4^+^ T cells after stimulation of total MLN cells with 50 ng/ml PMA and 250 ng/ml ionomycin. **(I)** Frequency of CD3^+^CD4^+^ T cells expressing Gata-3. **(J)** Dot plot showing gating on IL-4 producing CD4^+^ T cells by infected WT, B-IL-4^−/−^ and IL-4^−/−^ chimeras. Data are representative of two independent experiments. *n* = 4–6 mice.

Next, we investigated whether the lack of IL-4 expressing B cells affected the development of Th2 responses after *S. mansoni* infection. We stimulated total MLN cells from infected bone marrow chimeras with SEA or α-CD3 and detected cytokine production by ELISA from supernatants. Production of IL-4 was reduced in both B-IL-4^−/−^ and IL-4^−/−^ chimeras after either antigen-specific or mitogenic stimulation compared to WT controls (Figures 5A,B). Furthermore, the production of IL-10 was significantly decreased while the production of IL-17 was significantly increased both in B-IL-4^−/−^ and IL-4^−/−^ chimeras after α-CD3 restimulation compared to WT controls (Figure 5B). Finally, production of IFN-γ was increased in IL-4^−/−^ chimeras while it remained similar between B-IL-4^−/−^ and WT controls (Figure 5B).

We also analyzed intracellular cytokine secretion by B220^+^ B cells after restimulation of total MLN cells with PMA/ionomycin *ex vivo* and staining for flow cytometry analysis. The proportions of CD19^+^B220^+^ B cells was similar between all the chimeras (Figure 5C). Intracellular secretion of IL-4 by B220^+^ B cells from B-IL-4^−/−^ and IL-4^−/−^ chimeras was significantly reduced compared to WT chimeras (Figure 5D) while the levels of IFN-γ remained comparable between all the chimeras (Figure 5E). IL-4-producing B cells play a crucial role in the differentiation of Th2 cells *in vitro* (5, 7). Although we did not find a difference in the frequency of CD3^+^CD4^+^ T cells in chimeras lacking B cell-derived IL-4 (Figure 5F), the frequency of CD3^+^CD4^+^ T cells producing IL-4 (Figures 5G,J) and IFN-γ (Figure 5H) were comparable between B-IL-4^−/−^ and WT chimeras whereas the frequency of CD4^+^ T cells expressing Gata-3 (Figure 5I) was significantly reduced in B-IL-4^−/−^ chimeras compared to WT controls. The frequency of IL-4 (Figures 5G,J) and Gata-3 (Figure 5I) expressing CD3^+^CD4^+^ T cells was significantly reduced in IL-4^−/−^ chimeras where IFN-γ (Figure 5H) was unchanged compared to WT control chimeras. Therefore, these data indicate that IL-4 producing B cells are crucial for driving the development of Th2 responses during *S. mansoni* infection *in vivo*.

## Discussion

In this study, we examined the role of IL-4Rα expressing B cells in driving immunoregulation of inflammatory granulomatous tissue pathology during schistosomiasis. We found that mice carrying a specific deletion of IL-4Rα on B cells succumbed quickly to schistosomiasis compared to littermate control and B cell-deficient mice. Moreover, they failed to down-regulate lung granuloma size compared to littermate control mice during the *S. mansoni* egg challenge model. We also demonstrated that mice lacking IL-4Rα expressing B cells have enlarged liver and gut granulomas, and display a mixed cytokine profile indicated by augmented secretion of Th1, Th2, and Th17 cytokines at 24 weeks post-infection. Altogether, we could conclude that IL-4Rα expressing B cells are crucial for containment of excessive granulomatous tissue pathology and dampening of exuberant cytokine production during chronic schistosomiasis.

The contribution of B cells in driving host resistance to schistosomiasis is well established in the literature (1, 2). In fact, a study by Jankovic et al. demonstrated the importance of FcRγ expression on B cells in regulating excessive tissue pathology during both the acute and chronic phases of infection (1). In this study, we questioned whether a specific deletion of IL-4Rα expression on B cells could recapitulate the impaired host survival as a result of the general lack of B cells during *S. mansoni* infection. Interestingly, we found that B cell-specific IL-4Rα-deficient mice were highly susceptible to schistosomiasis suggesting that the lack of IL-4Rα expression on B cells, similarly to complete B-cell depletion sufficiently prevents the development of host protective immunity during schistosomiasis. These data suggest that B cells may be principally important in the regulation of tissue inflammation, whereas IL-4Rα expression on B cells may be crucial for the regulation of tissue inflammation and orchestrating host protective Th2 immunity. The observed delayed susceptibility of B cell deficient mice can potentially be explained by the 5-fold increased production of IL-10 by T cells after restimulation with α-CD3 compared to both B cell-specific IL-4Rα-deficient mice and littermate control mice. IL-10 has been implicated as a key immunoregulatory factor driving host resistance to schistosomiasis (23, 42). The fact that 50% of B cell deficient mice succumbed to disease at the beginning of the chronic stage of infection suggests that the mechanism of death may be different from that operating in B cell-specific IL-4Rα-deficient mice. Studies by Hoffman et al. and others have demonstrated that the mechanism of death can differ depending on the balance of the immune response, with mice having a skewed Th1 response succumbing early during infection due to cachexia and endotoxemia while mice with a skewed Th2 response die at the chronic stage of infection with increased fibrosis and large granulomas (22, 23, 32, 42).

The expansion and differentiation of cytokine producing T cells has been shown to be largely dependent on B cells (3, 7, 13). In our study, we found no significant difference in the absolute number of CD3^+^CD4^+^ T cells between all the mutant mouse strains. However, the expansion of follicular helper T cells (T_FH_) in the secondary lymphoid tissue was significantly impaired in *mb1*^cre^IL-4Rα^−/lox^ mice and μMT mice compared to littermate control mice. T_FH_ cells are crucial for lymphoid tissue organization by assisting in germinal center formation and promote antibody responses including isotype switching (43–45). In a study by Lin and colleagues, the deficiency of B cells was shown not to alter the differentiation of antigen-specific T cells and expression of activation markers CD69 and CD44 (7). Likewise, the expansion of effector CD4^+^ T cells was not hindered in B cell-specific IL-4Rα deficient mice whereas IL-4Rα^−/−^ mice displayed significantly reduced absolute numbers of effector CD4^+^ T cells compared to littermate control mice. Importantly, the lack of IL-4Rα expression on B cells resulted in abrogated intracellular production of Th2 cytokines IL-4 and IL-13 by CD4^+^ T cells after restimulation with PMA/Ionomycin *ex vivo*, suggesting that IL-4/IL-13 responsive B cells are crucial for promoting the expansion Th2 cells. Indeed, previous studies have shown that the lack of B cells alters the expansion and differentiation of IL-4 producing effector Th2 cells in response to *Hp* infection *in vivo* (3). Furthermore, B cells mediate the expansion of primary Th2 cells in response to protein antigens delivered with *Nippostrongylus brasiliensis* (*Nb*) (7) and protein antigens delivered with alum (4). B cells have also been shown to contribute to susceptibility during *Leishmania major* LV 39 infection in Balb/c mice and promote Th2 immunity (9).

Although there was no significant difference in the absolute number of CD19^+^B220^+^ B cells recruited into the MLN of infected mice, the number of follicular (FO) B cells was significantly reduced in both *mb1*^cre^IL-4Rα^−/lox^ compared to littermate control mice. Conversely, the number of marginal zone (MZ) B cells was significantly increased in B cell-specific IL-4Rα deficient mice compared to littermate control mice. FO B cells are found within germinal centers where they form tight physical contact with T cells, thus ensuring optimal T cell proliferation (46). B cells have been shown to produce Th2 cytokines in response to *Hp* infection *in vivo* (3, 13). The number of IL-4 and IL-13 producing B cells was reduced in both *mb1*^cre^IL-4Rα^−/lox^ and μMT mice after restimulation of total MLN cells with PMA/Ionomycin compared to the littermate control mice. Therefore, IL-4/IL-13 responsive B cells are important for maintaining optimal cellular immunity during infection with *S. mansoni*.

Down-modulation of the immune response and controlling the size of newly formed granulomas is essential for the host to survive chronic schistosomiasis (36, 37, 39, 47, 48). Earlier studies implicated CD8^+^ suppressor cells (36) and cross regulatory cytokines produced by CD4^+^ T cells (39, 49) in regulating exuberant host immune responses during the chronic stages of *S. mansoni* infection. However, a subsequent study by Yap et al. demonstrated a dispensable role for CD8^+^ T cells and IFN-γ in immunoregulation of tissue pathology during chronic schistosomiasis (47). We have recently shown that interfering with IL-4Rα signaling during the chronic phase of infection can ameliorate fibrogranulomatous pathology and reduce tissue scarring without being detrimental to host survival during chronic schistosomiasis (48). Of relevance to the current study, B cells have been shown to down-regulate granuloma formation without altering T cell responsiveness during chronic schistosomiasis (1, 2). A study by Fairfax and colleagues showed that blocking IL-10R resulted in the loss of B cells in the liver, consequently driving severe disease characterized by portosystemic shunting of the eggs to the heart and lungs during chronic schistosomiasis (41). Since we had established that deleting IL-4Rα expression on B cells impairs the development of type 2 instructing B effector cells, we next asked whether such cells were required for immunoregulation of fibrogranulomatous tissue inflammation during chronic schistosomiasis. We found that B cell-specific IL-4Rα-deficient mice had significantly larger granulomas at both 16 and 24 weeks post-infection compared to littermate control mice, suggesting that IL-4Rα expressing B cells are required to downregulate granulomatous pathology during chronic schistosomiasis. However, when comparing liver granuloma sizes of mb1^cre^IL-4Rα^−/lox^ mice between the two time points, we found that the granulomas at 24 weeks postinfection were smaller than those at 16 weeks post-infection. A similar trend was observed in littermate control mice, indicating the existence of a common immunomodulatory mechanism(s) still operating in the two strains. Interestingly, with the exception of IL-10, we found comparable cytokine responses between the two strains at 16 weeks post-infection. Such a reduction in IL-10 production tightly aligns with the high concentrations of α-CD3-driven IL-4, IL-5, IL-6, IL-10, IL-17, and IFN-γ that were observed at 24 weeks postinfection in the supernatants of total lymph node cell cultures from B cell-specific IL-4Rα-deficient mice when compared to those from littermate control mice. Tentatively, a case could be made for the importance of IL-4Rα expressing B cells in driving IL-10 production among other type-2 governed processes to ensure the control of acute granulomatous inflammation T cell responses and the resolution of excessive cytokine production during experimental schistosomiasis.

After establishing the requirement for IL-4Rα signaling on B cells in the development of IL-4 producing B cells and optimal Th2 responses to acute schistosomiasis, we questioned whether the development of optimal Th2 responses depends on both the ability of B cells to receive instruction from IL-4 and their ability to secrete IL-4 that triggers Th2 responses during *S. mansoni* infection. It had been previously shown that the lack of IL-4 producing B cells during *Hp* infection did not hamper parasite clearance, indicating that the development of protective immunity occurred independently of B cell-derived IL-4 (10). In contrast, using mixed bone marrow chimeras lacking B cell-derived IL-4 (B-IL-4^−/−^), we observed that secretion of IL-4 was significantly reduced after both antigen-specific stimulation and mitogenic stimulation with α-CD3 in cells from B-IL-4^−/−^ chimeras, similar to IL-4^−/−^ chimeras. Moreover, IL-10 was significantly reduced in cells from chimeras lacking B cell-derived IL-4 after mitogenic stimulation. This was later confirmed by analyzing intracellular cytokine production and transcription factor expression in CD4^+^ T cells, as we found significantly reduced frequencies of IL-4, IL-10, and Gata-3 expressing CD4^+^ T cells in B-IL-4^−/−^ chimeras compared to control WT chimeras. These results demonstrate that both the ability of B cells to receive instruction via IL-4Rα and B cell-derived IL-4 are essential for development of Th2 responses during acute schistosomiasis. These data corroborate our earlier findings, where we showed that chimeras lacking B cell-derived IL-4 had a skewed Th1 response characterized by up-regulation of the Th1 cytokine IFN-γ and down-regulation of the Th2 cytokines IL-4 and IL-13, that consequently rendered these mice resistant to *L. major* induced cutaneous leishmaniasis (9). Here, we have focused on B cell-derived IL-4, however, we cannot discount the involvement of other B cell-derived cytokines in driving development of Th2 immunity in response to helminth infections. In fact, a study by Wojciechowski et al. implicated B cell-derived TNF-α and IL-2 in mediating clearance of *Hp* infection, development of CD4^+^ T cells secreting IL-4 and secretion of protective type 2 antibody isotypes (10).

In summary, we have demonstrated that selective deletion of IL-4Rα on B cells renders mice more susceptible to acute schistosomiasis than B-cell deficient mice. We also unprecedentedly showed that IL-4Rα expressing B cells are required for immunoregulation of fibrogranulomatous tissue pathology and T cell responses during the late stages of chronic schistosomiasis. Our data therefore argue for the potential benefits of boosting IL-4Rα-mediated responses specifically on B cells to ameliorate fibrogranulomatous pathology associated with chronic schistosomiasis, especially in endemic areas.

## Materials and Methods

### Generation and Genotyping of *mb1*^cre^IL-4Rα^−/lox^ Balb/c Mice

*Mb1*^cre^ mice were intercrossed with IL-4Rα^lox/lox^ Balb/c mice (32, 50–52). These mice were further mated with homozygous IL-4Rα^−/−^ Balb/c mice (43) to generate hemizygous *mb1*^cre^IL-4Rα^−/lox^ mice (9). Hemizygous littermates (IL-4Rα^−/lox^) expressing functional IL-4Rα were used as wild-type controls in all experiments. Mice were genotyped as described previously (32, 43). All mice were housed in specific pathogen-free barrier conditions in individually ventilated cages at the University of Cape Town biosafety level 2 animal facility. Experimental mice were age and sex matched and used between 8 and 12 weeks of age.

### Ethics Statement

This study was performed in strict accordance with the recommendations of the South African national guidelines and University of Cape Town practice of laboratory animal procedure. All mouse experiments were performed according to the protocols approved by the Animal Research Ethics Committee of the Faculty of Health Sciences, University of Cape Town (protocol number: 010/041). Efforts were made to minimize and reduce suffering of animals.

### Live *S. Mansoni* Infection of Mice

Mice were percutaneously infected with 100 live cercariae (acute infection) or 30 live cercariae (chronic infection) that were provided by the Schistosome Research Reagent Resource Center for distribution by BEI Resources, (NIAID, NIH, USA). *Schistosoma mansoni*, Strain NMRI Exposed *Biomphalaria glabrata*, Strain NMRI, NR-21962. Mice were monitored weekly until the endpoint was reached (7 weeks for acute, 16 and 24 weeks for chronic schistosomiasis).

### Pulmonary *S. Mansoni* Eggs Model

Synchronous *S. mansoni* egg-challenge was conducted as previously described (44). Briefly, mice were sensitized to schistosome eggs by intraperitoneal injection of 2,500 eggs. Mice were subsequently challenged 14 days later by intravenous injection of 2,500 eggs and killed at day 7 and 14 post-challenge.

### Cell Preparation and *ex vivo* Restimulation

Single cell suspensions were prepared by pressing the draining lymph nodes through 70 μM cell-strainers. Cells were resuspended in complete IMDM (Gibco) supplemented with 10% FCS (Gibco) and penicillin and streptomycin (100 U/ml and 100 μg/ml, Gibco). The cells were cultured at 2 × 10^6^ cells/ml in 48-well plates coated with α-CD3 (20 μg/ml) or soluble egg antigen (SEA, 20 μg/ml) and incubated at 37°C in a humidified atmosphere containing 5% CO_2_. Supernatants were collected after 72 h and cytokines were measured by ELISA. Quantities of IL-4, IL-5, IL-10, IL-17, and IFN-γ were measured by sandwich ELISA as previously described (43).

### Antibodies and Flow Cytometry

The following antibodies comprising the B cell antibody panel were used: B220-V500, CD19-PerCP Cy5.5, CD23-PE, CD21-APC, CD24-PECy7, CD86-V450, MHCII-FITC, and IgM-Biotin (BD Bioscience, Erembodegem, Belgium). T cells panel consisted of the following antibodies: CD4-PerCP, CD3-AlexaFluor 700, CD62L-V500, CD44-FITC, CD28-PE, and CXCR5-V450 (BD Bioscience, Erembodegem, Belgium). Cells were acquired on a FACS Fortessa machine (BD Immunocytometry system, San Jose, CA, USA) and data was analyzed using Flowjo software (Treestar, Ashland, OR, USA).

### Intracellular Cytokine Staining

For detection of intracellular cytokines MST from *S. mansoni* eggs injected mice were plated at 2 × 10^6^ cells/well and stimulated at 37°C for 4 h with 50 ng/ml phorbal myristate acetate (PMA), 250 ng/ml ionomycin and 200 μM monensin in IMDM/10% FCS (all purchased from Sigma-Aldrich). Cells were stained with extracellular markers (CD4 Biotin-APC, or CD19 PercP), fixed for 30 min on ice in 2% (w/v) paraformaldehyde and permeabilised with 0.5% saponin buffer and stained with PE-labeled anti-mouse IL-4 and IL-10 for 30 min. Acquisition was performed using a FACSCalibur (BD Immunocytometry Systems, San Jose, CA, USA) and data were analyzed using FlowJo software (Treestar, Ashland, OR, USA).

### Enzyme Linked Immunosorbent Assays (ELISAs)

Cytokines in supernatant were measured by sandwich ELISA as previously described (43). For antibody ELISAs, blood was collected in serum separator tubes (BD Bioscience, San Diego, CA) and serum was separated by centrifugation at 8 000 × g for 10 min at 4°C. Titres of SEA-specific IgG1, IgG2a, IgG2b, and total IgE were determined as previously described (43).

### Hydroxyproline Assay

Hydroxyproline content as a measure of collagen production was determined using a modified protocol (45). Briefly, weighed liver samples were hydrolyzed and added to a 40 mg Dowex/Norit mixture. The supernatants were neutralized with 1% phenolphthalein and titrated against 10 M NaOH. An aliquot was mixed with isopropanol and added to chloramine-T/citrate buffer solution (pH 6.5). Erlich's reagent was added and absorbance was read at 570 nm. Hydroxyproline levels were calculated using 4-hydroxy-L-proline (Calbiochem) as a standard, and results were expressed as μmoles hydroxyproline per weight of tissue that contained 10^4^ eggs.

### Histology

Liver and gut samples were fixed in 4% (v/v) formaldehyde in phosphate buffered saline, embedded in wax and processed. Sections (5–7 μm) were stained with hematoxylin and eosin (H&E) and analine blue solution (CAB) and counterstained with Wegert's hematoxylin for collagen staining. Micrographs of liver granuloma were captured using a Nikon 5.0 mega pixel color digital camera (DCT DS-SMc).The diameter of each granuloma containing a single egg was measured with the ImageJ 1.34 software. An average of 25 granulomas per mouse was included in the analyses.

### Statistics

Statistical analysis was conducted using GraphPad Prism 4 software. Data was calculated as mean ± SD. Statistical significant was determined using the unpaired Student's *t*-test or 2-way ANOVA with Bonferroni's post test, defining differences to IL-4Rα^−/lox^ mice as significant (^*^p ≤ 0.05; ^**^p ≤ 0.01; ^***^p ≤ 0.001; Prism software; http://www.prism-software.com).

## Author Contributions

HN: Conceptualized the study, performed experiments, analyzed and interpreted data, acquired funding, wrote original manuscript and edited reviewed manuscript; JN: performed experiments, edited original manuscript and contributed in draft response to reviewers; NZ: Performed experiments, edited original manuscript and drafted response to reviewers; NN: Performed experiments, analyzed data, edited original manuscript and drafted response to reviewers; FB: Provided financial resources, supervision of the project and contributed in editing of the manuscript.

### Conflict of Interest Statement

The authors declare that the research was conducted in the absence of any commercial or financial relationships that could be construed as a potential conflict of interest.

## Supplementary Material

Figure S1 Reduced Th2 immunity in B cell-specific IL-4Rα-deficient mice. IL-4Rα^−/lox^, *mb1*^cre^IL-4Rα^−/lox^, and μMT mice were infected with 100 live *S. mansoni* cercariae and analyzed 7 weeks post-infection. Single cell suspension was prepared from mesenteric lymph node (MLN) and cells were stained for flow cytometry analysis. **(A)** Recruitment of CD3^+^CD4^+^ T cells into the secondary lymphoid tissue. **(B,C)** Expansion of CXCR5^+^ T_FH_ cells and effector CD4^+^ T cells (CD4^+^CD44^hi^CD62L^lo^) in the MLN. **(D)** Intracellular cytokine detection after restimulation of MLN cells with 50 ng/ml PMA and 250 ng/ml ionomycin *in vitro*. **(E–G)** Recruitment of CD19^+^B220^+^ B cells, follicular B cells (FO, B220^+^CD21^hi^CD23^hi^) and marginal zone B cells (MZ, B220^+^CD21^hi^CD23^lo^) into the secondary lymphoid tissue. **(H)** Analysis of intracellular cytokine production by CD19^+^ B cells after restimulation of total MLN cells with 50 ng/ml PMA and 250 ng/ml ionomycin *in vitro*. Data represents 3 independent experiments. *n* = 4–6 mice. ^*^*p* < 0.05, ^**^*p* < 0.01 and ^***^*p* < 0.001 vs. IL-4Rα^−/lox^ mice.

Figure S2 The absence of IL-4Rα responsive B cells leads to augmented gut inflammation in B cell-specific IL-4Rα-deficient mice during chronic schistosomiasis. IL-4Rα^−/lox^, *mb1*^cre^IL-4Rα^−/lox^ and IL-4Rα^−/lox^ mice were infected with 30 *S. mansoni* cercariae and analyzed at 16 and 24 weeks post-infection. Histological examination of gut tissue after staining sections with H&E (× 100). Data represent two independent experiments. *n* = 4–6 mice per group.

Figure S3 Comparable numbers of eggs in the lungs of infected mice at the chronic stages of schistosomiasis. IL-4Rα^−/lox^, *mb1*^cre^IL-4Rα^−/lox^ and IL-4Rα^−/lox^ mice were infected with 30 *S. mansoni* cercariae and analyzed at 16 and 24 weeks post-infection. Lungs were collected and the tissue was hydrolyzed overnight in 5% KOH and eggs were enumerated under a light microscope. **(A)** Egg numbers in the lungs at 16 weeks post-infection. **(B)** Egg numbers in the lungs at 24 weeks post-infection.

Figure S4 Gating strategy for B cells. Single cell suspensions were prepared from MLN and cells were stained for flow cytometry. Data was analyzed on FlowJo software and B cells were analyzed by gating on single cells, lymphocytes and CD19^+^B220^+^ B cells. CD21 and CD23 staining was used to delineate FO and MZ cells.

Figure S5 Gating strategy for CD4^+^ T cells. Single cell suspensions were prepared from MLN and cells were stained for flow cytometry. Data was analyzed on FlowJo software and CD4^+^ T cells were analyzed by gating on single cells, lymphocytes and CD3^+^CD4^+^ T cells. CD4^+^CD44^hi^CD62L^lo^ was used to delineate effector memory T cells and CD4^+^CXCR5^+^ T cells were T follicular helper (T_FH_) cells.

Figure S6 Schematic showing the generation of mixed bone marrow chimeras. Irradiated μMT mice were reconstituted 100% Balb/c BM (WT), 50% μMT and 50% IL-4^−/−^ BM (B-IL-4^−/−^) or 100% IL-4^−/−^ BM (IL-4^−/−^) and allowed to reconstitute for 8 weeks.

Figure S7 Successful reconstitution of bone marrow chimeras. Irradiated μMT mice were reconstituted 100% Balb/c BM (WT), 50% μMT and 50% IL-4^−/−^ BM (B-IL-4^−/−^) or 100% IL-4^−/−^ BM (IL-4^−/−^) and allowed to reconstitute for 8 weeks. Mice were bled at 8 weeks and cells were stained for flow cytometry analysis. **(A)** Proportions of CD3^+^CD4^+^ T cells in peripheral blood after reconstitution. **(B)** Proportions of CD19^+^B220^+^ B cells found in blood after reconstitution. **(C)** Frequency of CD11b^+^ cells in peripheral blood. **(D)** Frequency of CD11c^+^ cells found in peripheral blood after reconstitution of bone marrow chimeras. Data represent two independent experiments. *n* = 6 mice per group.

Figure S8 Sufficient humoral immunity develops in mice lacking IL-4 producing B cells during *S. mansoni* infection. Irradiated μMT mice were reconstituted with 100% Balb/c bone marrow cells (WT), 50% μMT and 50% IL-4^−/−^ bone marrow cells (B-IL-4^−/−^) or 100% IL-4^−/−^ bone marrow cells (IL-4^−/−^) and infected with 100 *S. mansoni* cercariae. Mice were killed 7 weeks post-infection and blood was collected for serum separation. **(A–D)** Serum antibody titers detected by ELISA. Data represent two independent experiments. ^*^*p* < 0.05 vs. WT mice. *n* = 4–6 mice per group.

Table S1 Percentage of mice that died during the course of the chronic schistosomiasis. Mice were infected with 30 live *S. mansoni* cercariae and killed at 16 and 24 weeks post-infection.

## References

[1] Jankovic D Cheever AW Kullberg MC Wynn TA Yap G Caspar P. CD4+ T cell-mediated granulomatous pathology in schistosomiasis is downregulated by a B cell-dependent mechanism requiring Fc receptor signaling. J Exp Med. (1998) 187:619–29. 10.1084/jem.187.4.619 9463412 PMC2212140

[2] Ferru I Roye O Delacre M Auriault C Wolowczuk I. Infection of B-cell-deficient mice by the parasite *Schistosoma mansoni*: demonstration of the participation of B cells in granuloma modulation. Scand J Immunol. (1998) 48:233–40. 10.1046/j.1365-3083.1998.00376.x 9743206

[3] Lund FE Hollifield M Schuer K Lines JL Randall TD Garvy BA. B cells are required for generation of protective effector and memory CD4 cells in response to Pneumocystis lung infection. J Immunol. (2006) 176:6147–54. 10.4049/jimmunol.176.10.6147 16670323

[4] Ronet C Voigt H Himmelrich H Doucey M-A Hauyon-La Torre Y Revaz-Breton M. Leishmania major-specific B cells are necessary for Th2 cell development and susceptibility to L. major LV39 in BALB/c mice. J Immunol. (2008) 180:4825–35. 10.4049/jimmunol.180.7.4825 18354206

[5] Harris DP Haynes L Sayles PC Duso DK Eaton SM Lepak NM. Reciprocal regulation of polarized cytokine production by effector B and T cells. Nat Immunol. (2000) 1:475–82. 10.1038/82717 11101868

[6] Harris DP Goodrich S Gerth AJ Peng SL Lund FE. Regulation of IFN-gamma production by B effector 1 cells: essential roles for T-bet and the IFN-gamma receptor. J Immunol. (2005) 174:6781–90. 10.4049/jimmunol.174.11.6781 15905519

[7] Harris DP Goodrich S Mohrs K Mohrs M Lund FE. Cutting edge: the development of IL-4-producing B cells (B effector 2 cells) is controlled by IL-4, IL-4 receptor alpha, and Th2 cells. J Immunol. (2005) 175:7103–7. 10.4049/jimmunol.175.11.7103 16301612

[8] Lund FE. Cytokine-producing B lymphocytes—key regulators of immunity. Curr Opin Immunol. (2008) 20:332–8. 10.1016/j.coi.2008.03.003 18417336 PMC2474694

[9] Hurdayal R Ndlovu HH Revaz-Breton M Parihar SP Nono JK Govender M. IL-4–producing B cells regulate T helper cell dichotomy in type 1- and type 2-controlled diseases. Proc Natl Acad Sci USA. (2017) 114:E8430–9. 10.1073/pnas.1708125114 28916732 PMC5635893

[10] Wojciechowski W Harris DP Sprague F Mousseau B Makris M Kusser K. Cytokine-producing effector B cells regulate type 2 immunity to H. polygyrus. Immunity (2009) 30:421–33. 10.1016/j.immuni.2009.01.006 19249230 PMC2745290

[11] Colley DG Bustinduy AL Secor WE King CH. Human schistosomiasis. Lancet (2014) 383:2253–64. 10.1016/S0140-6736(13)61949-2 24698483 PMC4672382

[12] Gryseels B Polman K Clerinx J Kestens L. Human schistosomiasis. Lancet (2006) 368:1106–18. 10.1016/S0140-6736(06)69440-3 16997665

[13] Ross AGP Bartley PB Sleigh AC Olds GR Li Y Williams GM. Schistosomiasis. N Engl J Med. (2002) 346:1212–20. 10.1056/NEJMra012396 11961151

[14] van der Werf MJ de Vlas SJ Brooker S Looman CWN Nagelkerke NJD Habbema JD. Quantification of clinical morbidity associated with schistosome infection in sub-Saharan Africa. Acta Trop. 86:125–39. 10.1016/S0001-706X(03)00029-9 12745133

[15] Pearce EJ MacDonald AS. The immunobiology of schistosomiasis. Nature Rev Immunol. (2002) 2:499–511. 10.1038/nri843 12094224

[16] Ndlovu H Brombacher F. Role of IL-4Rα during acute schistosomiasis in mice. Parasite Immunol. (2013) 36:421–7. 10.1111/pim.12080 24127774 PMC4286023

[17] Fine DP Buchanan RD Colley DG *Schistosoma mansoni* infection in mice depleted of thymus-dependent lymphocytes. I. Eosinophilia and immunologic responses to a schistosomal egg preparation. Am J Pathol. (1973) 71:193–206. 4541345 PMC1903959

[18] Buchanan RD Fine DP Colley DG *Schistosoma mansoni* infection in mice depleted of thymus-dependent lymphocytes. II. Pathology and altered pathogenesis. Am J Pathol. (1973) 71:207–18. 4541346 PMC1903958

[19] Byram JE von Lichtenberg F. Altered schistosome granuloma formation in nude mice. Am J Trop Med Hyg. (1977) 26:944–56. 10.4269/ajtmh.1977.26.944 303056

[20] Byram JE Doenhoff MJ Musallam R Brink LH von Lichtenberg F. *Schistosoma mansoni* infections in T-cell deprived mice, and the ameliorating effect of administering homologous chronic infection serum. II. Pathology. Am J Trop Med Hyg. (1979) 28:274–85. 10.4269/ajtmh.1979.28.274 313162

[21] Doenhoff M Musallam R Bain J McGregor A *Schistosoma mansoni* infections in T-cell deprived mice, and the ameliorating effect of administering homologous chronic infection serum. I. Pathogenesis. Am J Trop Med Hyg. (1979) 28:260–3. 10.4269/ajtmh.1979.28.260 313161

[22] Brunet LR Finkelman FD Cheever AW Kopf MA Pearce EJ. IL-4 protects against TNF-alpha-mediated cachexia and death during acute schistosomiasis. J Immunol. (1997) 159:777–85. 9218595

[23] Hoffmann KF Cheever AW Wynn TA. IL-10 and the dangers of immune polarization: excessive type 1 and type 2 cytokine responses induce distinct forms of lethal immunopathology in murine schistosomiasis. J Immunol. (2000) 164:6406–16. 10.4049/jimmunol.164.12.6406 10843696

[24] Chiaramonte MG Schopf LR Neben TY Cheever AW Donaldson DD Wynn TA. IL-13 is a key regulatory cytokine for Th2 cell-mediated pulmonary granuloma formation and IgE responses induced by *Schistosoma mansoni* eggs. J Immunol. (1999) 162:920–30. 9916716

[25] Chiaramonte MG Donaldson DD Cheever AW Wynn TA. An IL-13 inhibitor blocks the development of hepatic fibrosis during a T-helper type 2-dominated inflammatory response. J Clin Invest. (1999) 104:777–85. 10.1172/JCI7325 10491413 PMC408441

[26] Pearce EJ Cheever A Leonard S Covalesky M Fernandez-Botran R Kohler G. *Schistosoma mansoni* in IL-4-deficient mice. Inter Immunol. (1996) 8:435–44. 8671630 10.1093/intimm/8.4.435

[27] Fallon PG Richardson EJ McKenzie GJ McKenzie AN. Schistosome infection of transgenic mice defines distinct and contrasting pathogenic roles for IL-4 and IL-13: IL-13 is a profibrotic agent. J Immunol. (2000) 164:2585–91. 10.4049/jimmunol.164.5.2585 10679097

[28] Jankovic D Kullberg MC Noben-Trauth N Caspar P Paul WE Sher A. Single cell analysis reveals that IL-4 receptor/Stat6 signaling is not required for the *in vivo* or *in vitro* development of CD4+ lymphocytes with a Th2 cytokine profile. J Immunol. (2000) 164:3047–55. 10.4049/jimmunol.164.6.3047 10706693

[29] Jankovic D Kullberg MC Noben-Trauth N Caspar P Ward JM Cheever AW. Schistosome-infected IL-4 receptor knockout (KO) mice, in contrast to IL-4 KO mice, fail to develop granulomatous pathology while maintaining the same lymphokine expression profile. J Immunol. (1999) 163:337–42. 10384133

[30] Kaplan MH Whitfield JR Boros DL Grusby MJ. Th2 cells are required for the *Schistosoma mansoni* egg-induced granulomatous response. J Immunol. (1998) 160:1850–56. 9469446

[31] Nono JK Ndlovu H Abdel Aziz N Mpotje T Hlaka L Brombacher F. Interleukin-4 receptor alpha is still required after Th2 polarization for the maintenance and the recall of protective immunity to Nematode infection. PLoS Negl Trop Dis. (2017) 11:e0005675. 10.1371/journal.pntd.0005675 28651009 PMC5501681

[32] Herbert DR Hölscher C Mohrs M Arendse B Schwegmann A Radwanska M. Alternative macrophage activation is essential for survival during schistosomiasis and downmodulates T helper 1 responses and immunopathology. Immunity (2004) 20:623–35. 10.1016/S1074-7613(04)00107-4 15142530

[33] Marillier RG Brombacher TM Dewals B Leeto M Barkhuizen M Govender D. IL-4R alpha responsive smooth muscle cells increase intestinal hypercontractility and contribute to resistance during acute Schistosomiasis. Am J Physiol Gastrointest Liver Physiol. (2010) 298:G943–51. 10.1152/ajpgi.00321.2009 20360135

[34] Dewals B Hoving JC Leeto M Marillier RG Govender U Cutler AJ IL-4R alpha responsiveness of non-CD4 T cells contributes to resistance in schistosoma mansoni infection in pan-T cell-specific IL-4Ralpha-deficient mice. Am J Pathol. (2009) 175:706–16. 10.2353/ajpath.2009.090137 19628763 PMC2716945

[35] Cheever AW Jankovic D Yap GS Kullberg MC Sher A Wynn TA Role of cytokines in the formation and downregulation of hepatic circumoval granulomas and hepatic fibrosis in *Schistosoma mansoni*-infected mice. Mem Inst Oswaldo Cruz. (1998) 93 Suppl 1:25–32. 10.1590/s0074-02761998000700004 9921320

[36] Chensue SW Warmington KS Hershey SD Terebuh PD Othman M Kunkel SL. Evolving T cell responses in murine schistosomiasis. Th2 cells mediate secondary granulomatous hypersensitivity and are regulated by CD8+ T cells in vivo. J Immunol. (1993) 151:1391–400. 8335935

[37] Chensue SW Warmington KS Ruth J Lincoln PM Kunkel SL. Cross-regulatory role of interferon-gamma (IFN-gamma), IL-4 and IL-10 in schistosome egg granuloma formation: *in vivo* regulation of Th activity and inflammation. Clin Exp Immunol. (1994) 98:395–400. 10.1111/j.1365-2249.1994.tb05503.x 7994903 PMC1534488

[38] Flores-Villanueva PO Zheng XX Strom TB Stadecker MJ. Recombinant IL-10 and IL-10/Fc treatment down-regulate egg antigen-specific delayed hypersensitivity reactions and egg granuloma formation in schistosomiasis. J Immunol. (1996) 156:3315–20. 8617955

[39] Bosshardt SC Freeman GL Secor WE Colley DG. IL-10 deficit correlates with chronic, hypersplenomegaly syndrome in male CBA/J mice infected with *Schistosoma mansoni*. Parasite Immunol. (1997) 19:347–53. 10.1046/j.1365-3024.1997.d01-224.x 9292893

[40] Sadler CH Rutitzky LI Stadecker MJ Wilson RA. IL-10 is crucial for the transition from acute to chronic disease state during infection of mice with *Schistosoma mansoni*. Eur J Immunol. (2003) 33:880–8. 10.1002/eji.200323501 12672053

[41] Fairfax KC Amiel E King IL Freitas TC Mohrs M Pearce EJ. IL-10R blockade during chronic *Schistosomiasis mansoni* results in the loss of B cells from the liver and the development of severe pulmonary disease. PLoS Pathog. (2012) 8:e1002490. 10.1371/journal.ppat.1002490 22291593 PMC3266936

[42] Herbert DR Orekov T Perkins C Finkelman FD. IL-10 and TGF-beta redundantly protect against severe liver injury and mortality during acute schistosomiasis. J Immunol. (2008) 181:7214–20. 10.4049/jimmunol.181.10.7214 18981143 PMC2921214

[43] Mohrs M Ledermann B Köhler G Dorfmüller A Gessner A Brombacher F. Differences between IL-4- and IL-4 receptor alpha-deficient mice in chronic leishmaniasis reveal a protective role for IL-13 receptor signaling. J Immunol. (1999) 162:7302–8. 10358179

[44] Wynn TA Eltoum I Cheever AW Lewis FA Gause WC Sher A. Analysis of cytokine mRNA expression during primary granuloma formation induced by eggs of *Schistosoma mansoni*. J Immunol. (1993) 151:1430–40. 8335939

[45] Bergman I Loxley R Two improved and simplified methods for the spectrophotometric determination of hydroxyproline. Anal Chem. (1963) 35:1961–5. 10.1021/ac60205a053

[46] Garside P Ingulli E Merica RR Johnson JG Noelle RJ Jenkins MK. Visualization of specific B and T lymphocyte interactions in the lymph node. (1998) Science 281:96–9. 10.1126/science.281.5373.96 9651253

[47] Yap G Cheever A Caspar P Jankovic D Sher A. Unimpaired down-modulation of the hepatic granulomatous response in CD8 T-cell- and gamma interferon-deficient mice chronically infected with *Schistosoma mansoni*. Infect Immun. (1997) 65:2583–6. 9199423 10.1128/iai.65.7.2583-2586.1997 PMC175365

[48] Nono JK Ndlovu H Aziz NA Mpotje T Hlaka L Brombacher F. Host regulation of liver fibroproliferative pathology during experimental schistosomiasis via interleukin-4 receptor alpha. PLoS Negl Trop Dis. (2017) 11:e0005861. 10.1371/journal.pntd.0005861 28827803 PMC5578697

[49] Flores-Villanueva PO Zheng XX Strom TB Stadecker MJ. Recombinant IL-10 and IL-10/Fc treatment down-regulate egg antigen-specific delayed hypersensitivity reactions and egg granuloma formation in schistosomiasis. J Immunol. (1996) 156:3315–20. 8617955

[50] Hoving JC Kirstein F Nieuwenhuizen NE Fick LCE Hobeika E Reth M. B Cells that produce immunoglobulin E mediate colitis in BALB/c mice. Gastroenterology (2012) 142:96–108. 10.1053/j.gastro.2011.09.044 21983080

[51] Hobeika E Thiemann S Storch B Jumaa H Nielsen PJ Pelanda R. Testing gene function early in the B cell lineage in mb1-cre mice. Proc Natl Acad Sci USA. (2006) 103:13789–94. 10.1073/pnas.0605944103 16940357 PMC1564216

[52] Reth M Wienands J. Initiation and processing of signals from the B cell antigen receptor. Annu Rev Immunol. (1997) 15:453–79. 10.1146/annurev.immunol.15.1.453 9143696

