# Supplementary figures and images for: Interleukin-4 Receptor Alpha Expressing B Cells Are Essential to Down-Modulate Host Granulomatous Inflammation During Schistosomasis
Source: Front Immunol. 2018 Dec 18;9:2928. doi: 10.3389/fimmu.2018.02928 (PMC6305417; doi:10.3389/fimmu.2018.02928)

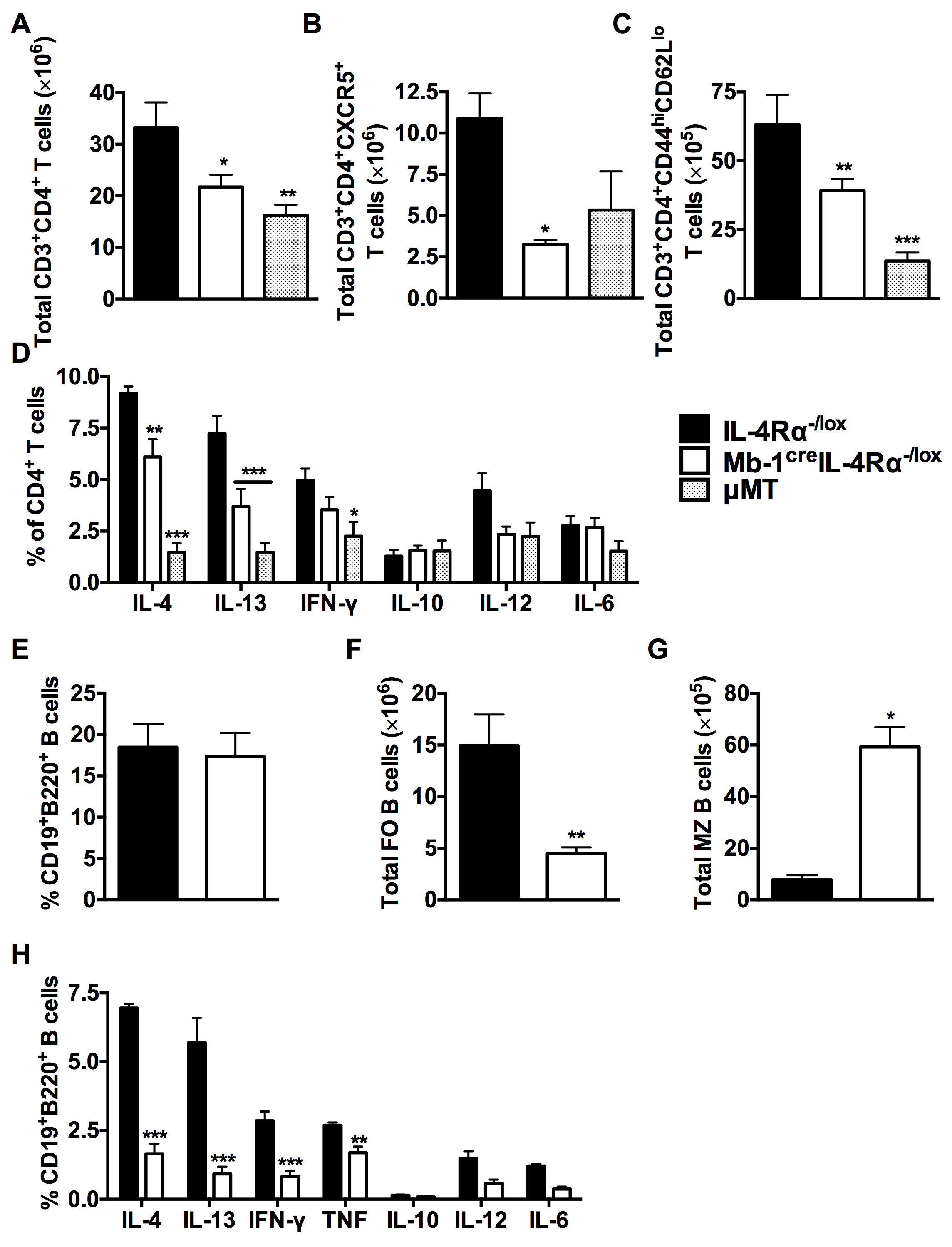

Supplement: Figure S1 — Reduced Th2 immunity in B cell-specific IL-4Rα-deficient mice. IL-4Rα−/lox, mb1creIL-4Rα−/lox, and μMT mice were infected with 100 live S. mansoni cercariae and analyzed 7 weeks post-infection. Single cell suspension was prepared from mesenteric lymph node (MLN) and cells were stained for flow cytometry analysis. (A) Recruitment of CD3+CD4+ T cells into the secondary lymphoid tissue. (B,C) Expansion of CXCR5+ TFH cells and effector CD4+ T cells (CD4+CD44hiCD62Llo) in the MLN. (D) Intracellular cytokine detection after restimulation of MLN cells with 50 ng/ml PMA and 250 ng/ml ionomycin in vitro. (E–G) Recruitment of CD19+B220+ B cells, follicular B cells (FO, B220+CD21hiCD23hi) and marginal zone B cells (MZ, B220+CD21hiCD23lo) into the secondary lymphoid tissue. (H) Analysis of intracellular cytokine production by CD19+ B cells after restimulation of total MLN cells with 50 ng/ml PMA and 250 ng/ml ionomycin in vitro. Data represents 3 independent experiments. n = 4–6 mice. *p < 0.05, **p < 0.01 and ***p < 0.001 vs. IL-4Rα−/lox mice. [file Image_1.TIFF]

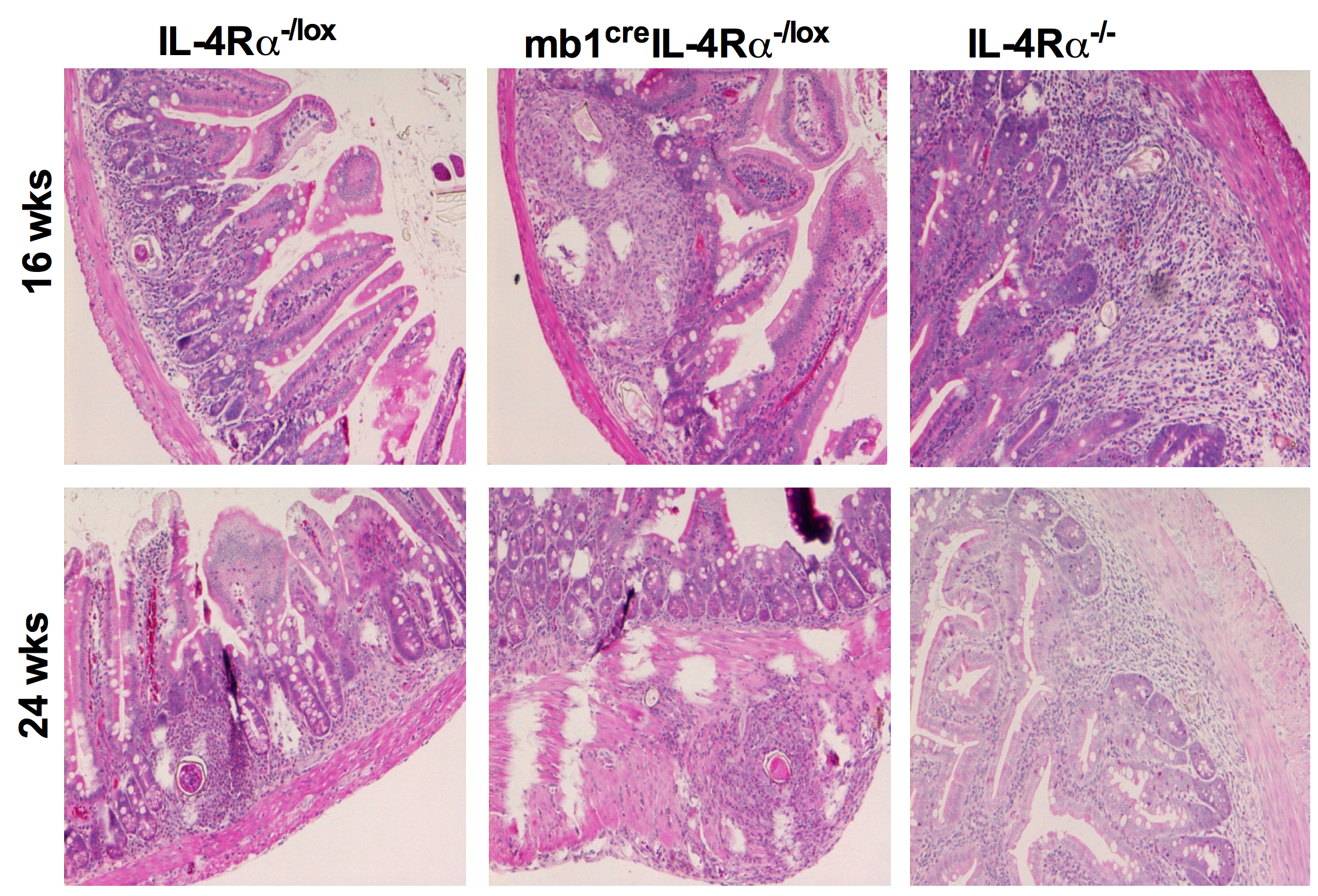

Supplement: Figure S2 — The absence of IL-4Rα responsive B cells leads to augmented gut inflammation in B cell-specific IL-4Rα-deficient mice during chronic schistosomiasis. IL-4Rα−/lox, mb1creIL-4Rα−/lox and IL-4Rα−/lox mice were infected with 30 S. mansoni cercariae and analyzed at 16 and 24 weeks post-infection. Histological examination of gut tissue after staining sections with H&E (× 100). Data represent two independent experiments. n = 4–6 mice per group. [file Image_2.TIFF]

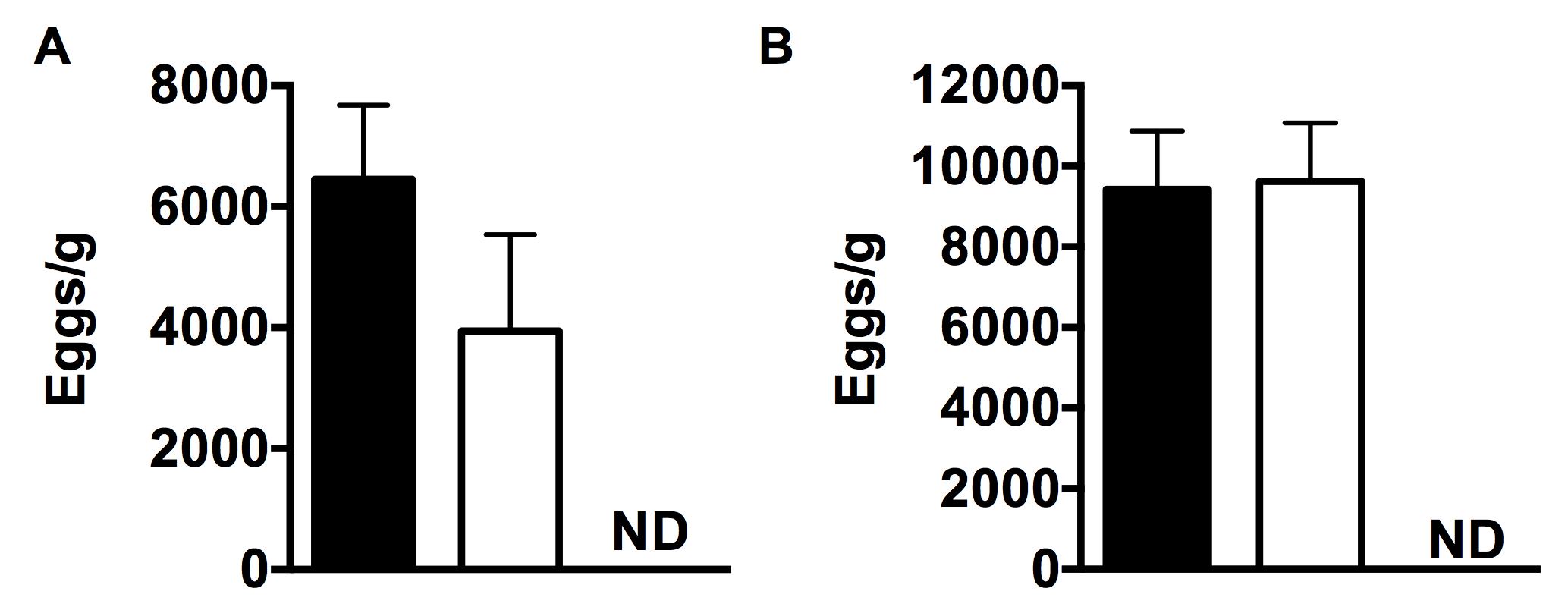

Supplement: Figure S3 — Comparable numbers of eggs in the lungs of infected mice at the chronic stages of schistosomiasis. IL-4Rα−/lox, mb1creIL-4Rα−/lox and IL-4Rα−/lox mice were infected with 30 S. mansoni cercariae and analyzed at 16 and 24 weeks post-infection. Lungs were collected and the tissue was hydrolyzed overnight in 5% KOH and eggs were enumerated under a light microscope. (A) Egg numbers in the lungs at 16 weeks post-infection. (B) Egg numbers in the lungs at 24 weeks post-infection. [file Image_3.TIFF]

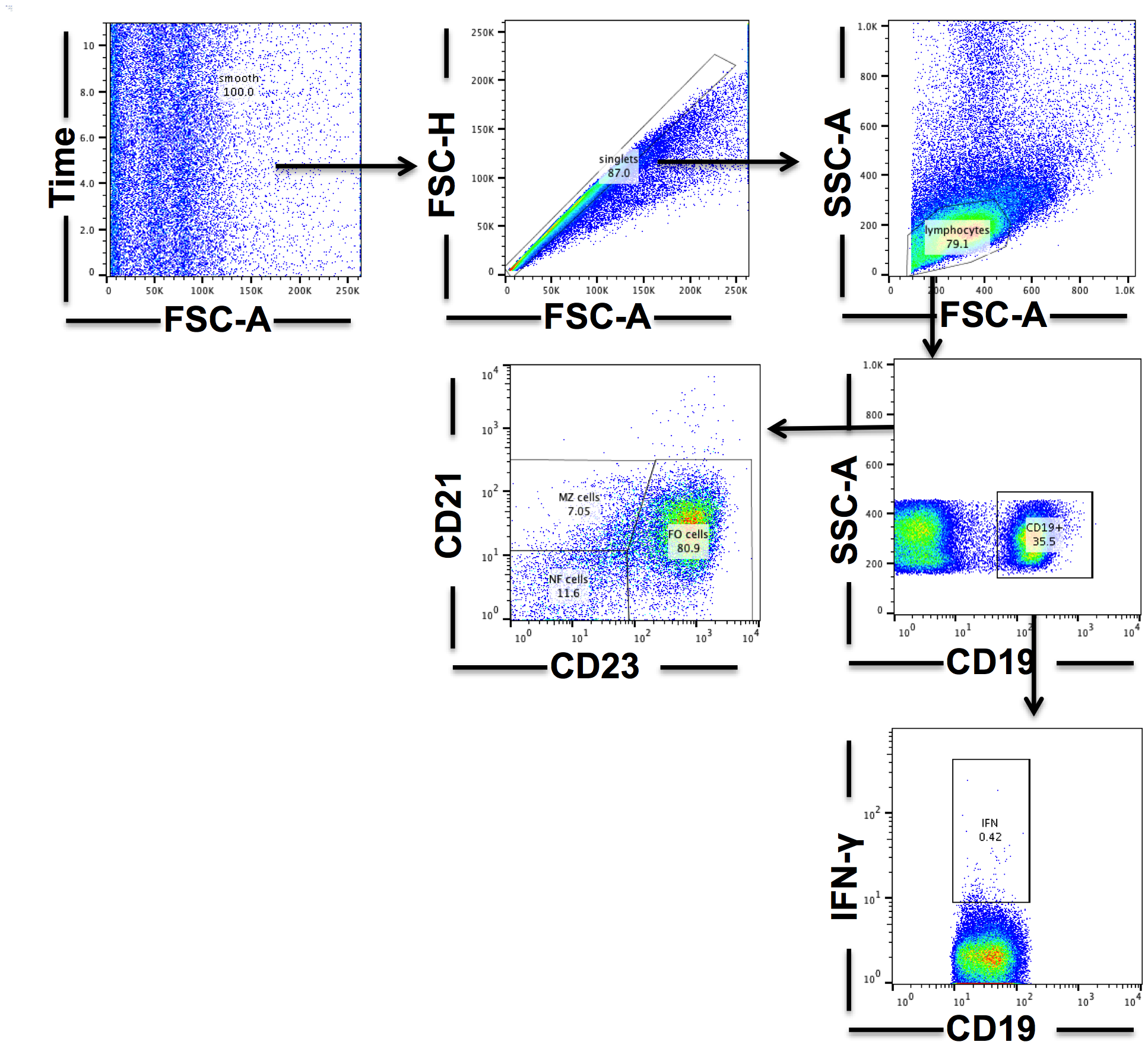

Supplement: Figure S4 — Gating strategy for B cells. Single cell suspensions were prepared from MLN and cells were stained for flow cytometry. Data was analyzed on FlowJo software and B cells were analyzed by gating on single cells, lymphocytes and CD19+B220+ B cells. CD21 and CD23 staining was used to delineate FO and MZ cells. [file Image_4.TIFF]

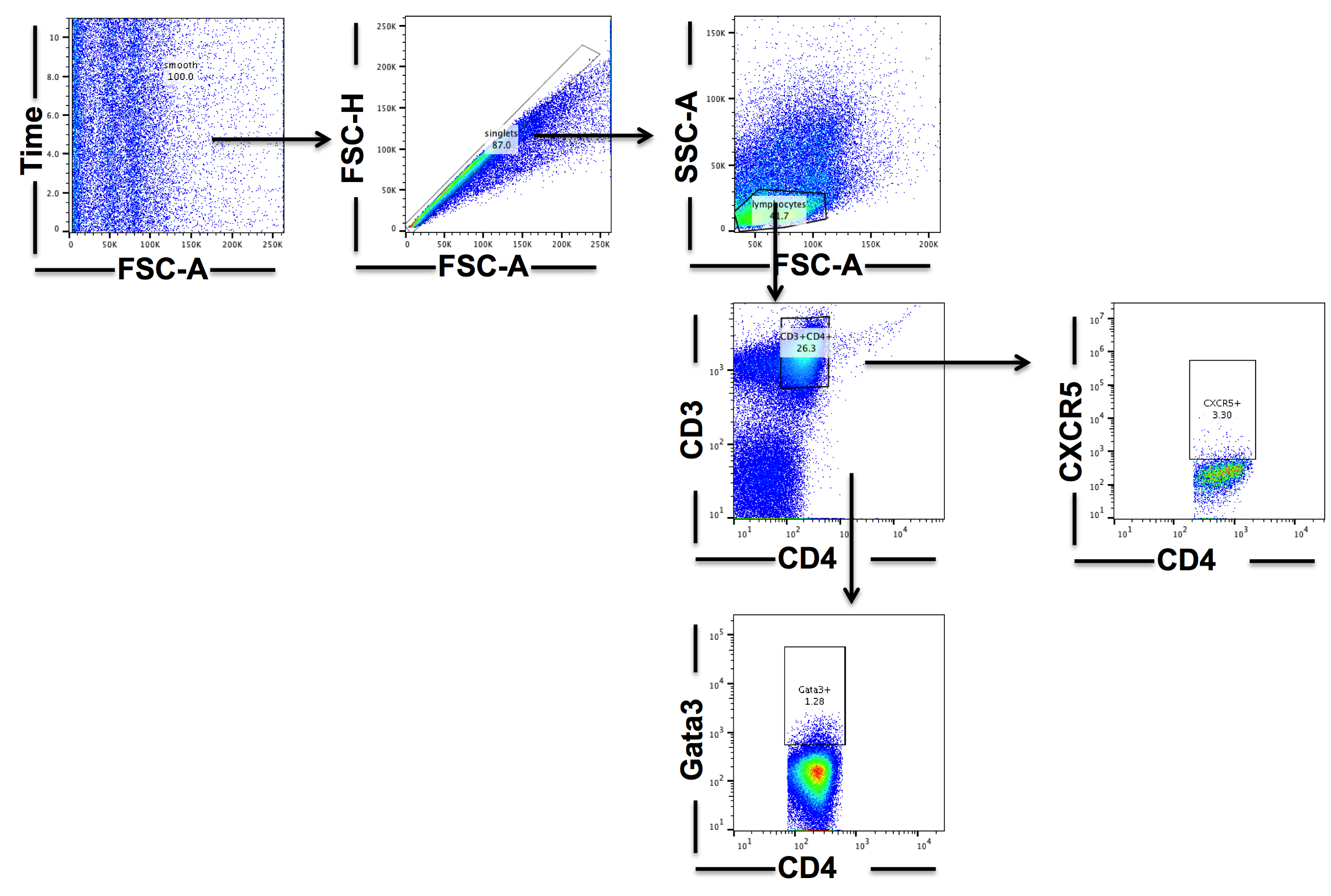

Supplement: Figure S5 — Gating strategy for CD4+ T cells. Single cell suspensions were prepared from MLN and cells were stained for flow cytometry. Data was analyzed on FlowJo software and CD4+ T cells were analyzed by gating on single cells, lymphocytes and CD3+CD4+ T cells. CD4+CD44hiCD62Llo was used to delineate effector memory T cells and CD4+CXCR5+ T cells were T follicular helper (TFH) cells. [file Image_5.TIFF]

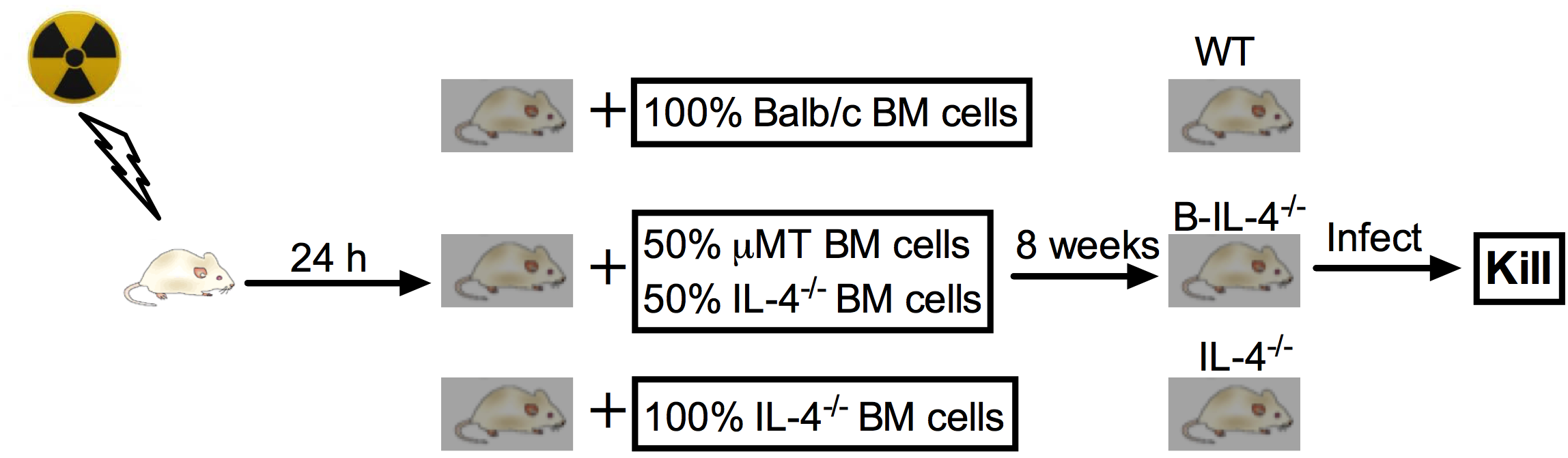

Supplement: Figure S6 — Schematic showing the generation of mixed bone marrow chimeras. Irradiated μMT mice were reconstituted 100% Balb/c BM (WT), 50% μMT and 50% IL-4−/− BM (B-IL-4−/−) or 100% IL-4−/− BM (IL-4−/−) and allowed to reconstitute for 8 weeks. [file Image_6.TIFF]

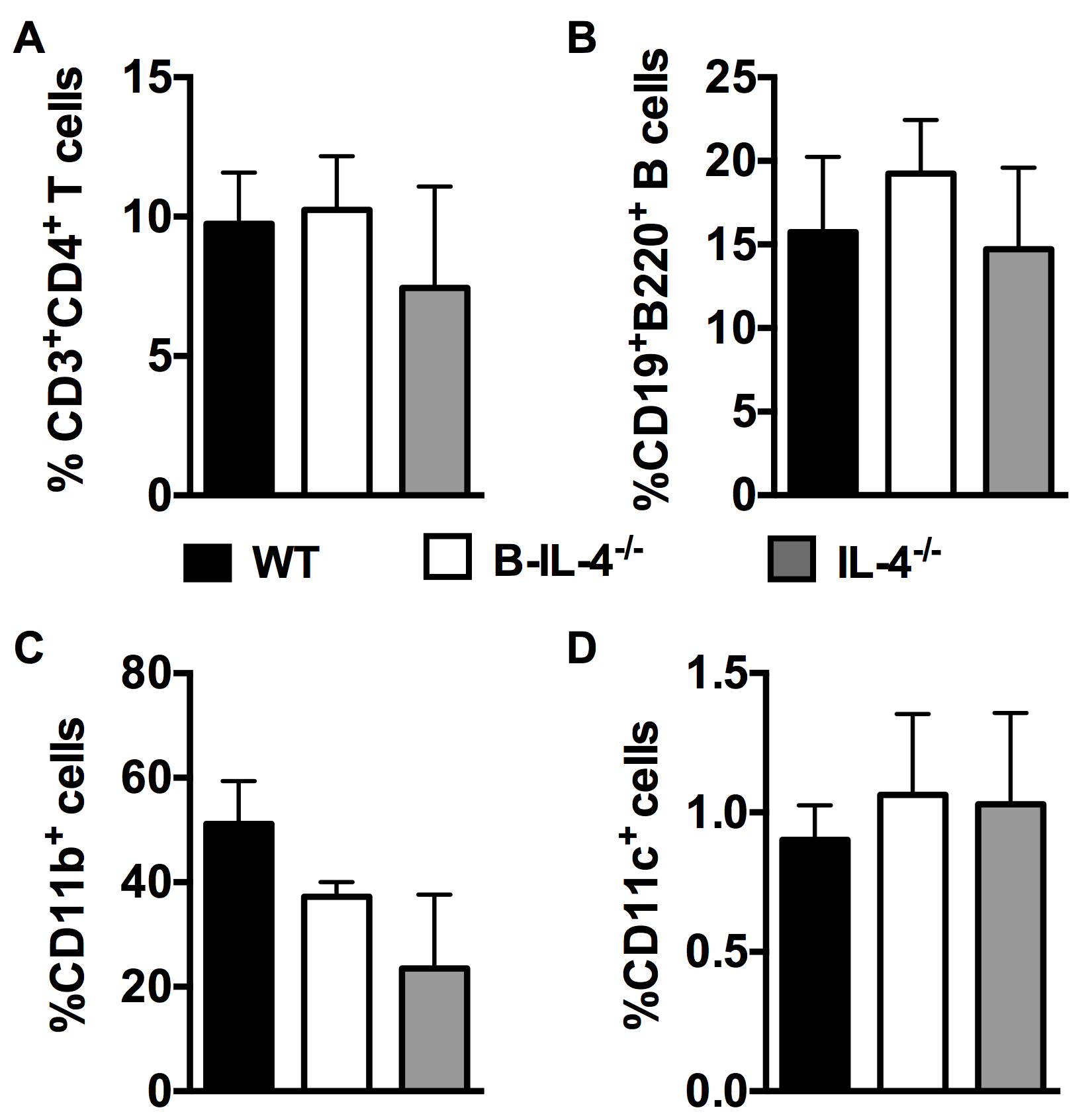

Supplement: Figure S7 — Successful reconstitution of bone marrow chimeras. Irradiated μMT mice were reconstituted 100% Balb/c BM (WT), 50% μMT and 50% IL-4−/− BM (B-IL-4−/−) or 100% IL-4−/− BM (IL-4−/−) and allowed to reconstitute for 8 weeks. Mice were bled at 8 weeks and cells were stained for flow cytometry analysis. (A) Proportions of CD3+CD4+ T cells in peripheral blood after reconstitution. (B) Proportions of CD19+B220+ B cells found in blood after reconstitution. (C) Frequency of CD11b+ cells in peripheral blood. (D) Frequency of CD11c+ cells found in peripheral blood after reconstitution of bone marrow chimeras. Data represent two independent experiments. n = 6 mice per group. [file Image_7.TIFF]

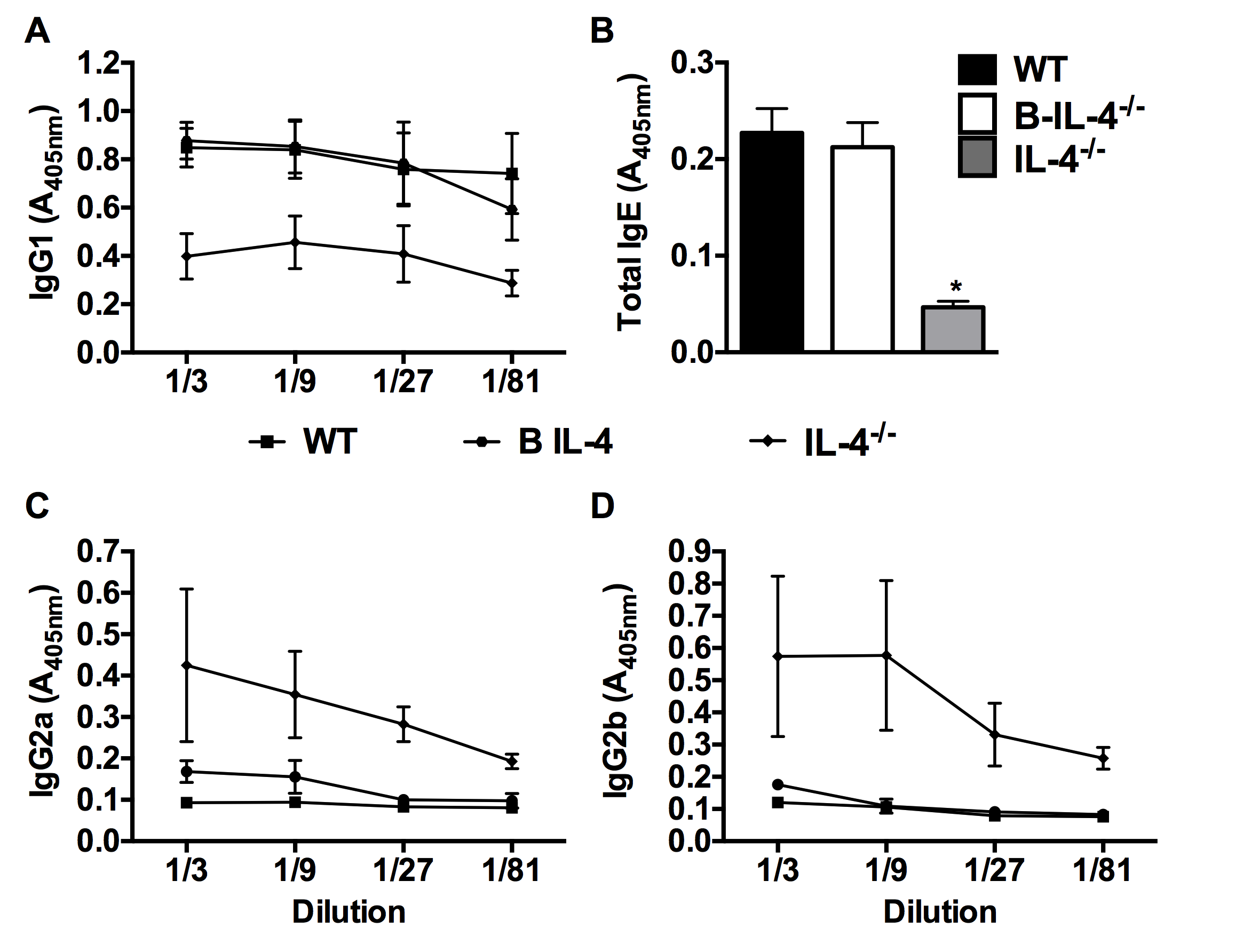

Supplement: Figure S8 — Sufficient humoral immunity develops in mice lacking IL-4 producing B cells during S. mansoni infection. Irradiated μMT mice were reconstituted with 100% Balb/c bone marrow cells (WT), 50% μMT and 50% IL-4−/− bone marrow cells (B-IL-4−/−) or 100% IL-4−/− bone marrow cells (IL-4−/−) and infected with 100 S. mansoni cercariae. Mice were killed 7 weeks post-infection and blood was collected for serum separation. (A–D) Serum antibody titers detected by ELISA. Data represent two independent experiments. *p < 0.05 vs. WT mice. n = 4–6 mice per group. [file Image_8.TIFF]

**Table S1:**


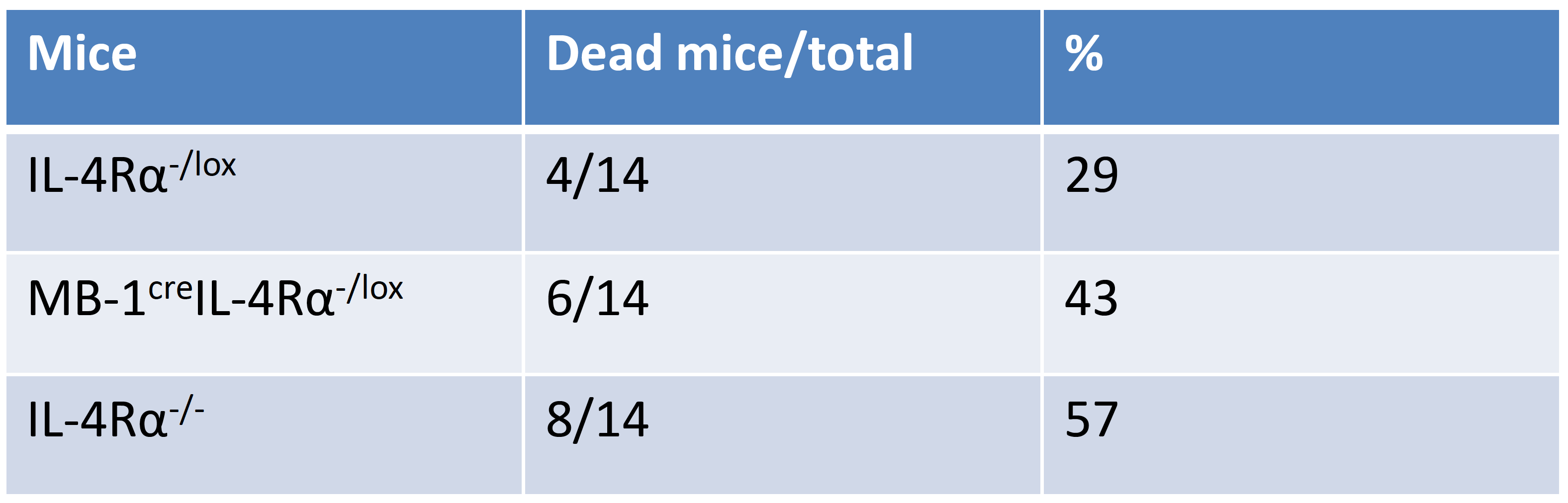

Supplement: Table S1 — Percentage of mice that died during the course of the chronic schistosomiasis. Mice were infected with 30 live S. mansoni cercariae and killed at 16 and 24 weeks post-infection. [file Table_1.DOCX]
